# Understanding practice: the factors that influence management of mild traumatic brain injury in the emergency department-a qualitative study using the Theoretical Domains Framework

**DOI:** 10.1186/1748-5908-9-8

**Published:** 2014-01-13

**Authors:** Emma J Tavender, Marije Bosch, Russell L Gruen, Sally E Green, Jonathan Knott, Jill J Francis, Susan Michie, Denise A O’Connor

**Affiliations:** 1 National Trauma Research Institute, The Alfred Hospital/Department of Surgery, Monash University, Melbourne, Australia; 2 Department of Trauma, The Alfred Hospital/Department of Surgery, Monash University/National Trauma Research Institute, Melbourne, Australia; 3 School of Public Health and Preventative Medicine, Monash University, Melbourne, Australia; 4 Melbourne Medical School and Department of Emergency Medicine, Royal Melbourne Hospital, Melbourne, Australia; 5 School of Health Sciences, City University, London, UK; 6 Department of Clinical, Educational and Health Psychology, University College London, London, UK

**Keywords:** Emergency department management, Mild traumatic brain injury, Theoretical Domains Framework, Semi-structured interviews

## Abstract

**Background:**

Mild traumatic brain injury is a frequent cause of presentation to emergency departments. Despite the availability of clinical practice guidelines in this area, there is variation in practice. One of the aims of the Neurotrauma Evidence Translation program is to develop and evaluate a targeted, theory- and evidence-informed intervention to improve the management of mild traumatic brain injury in Australian emergency departments. This study is the first step in the intervention development process and uses the Theoretical Domains Framework to explore the factors perceived to influence the uptake of four key evidence-based recommended practices for managing mild traumatic brain injury.

**Methods:**

Semi-structured interviews were conducted with emergency staff in the Australian state of Victoria. The interview guide was developed using the Theoretical Domains Framework to explore current practice and to identify the factors perceived to influence practice. Two researchers coded the interview transcripts using thematic content analysis.

**Results:**

A total of 42 participants (9 Directors, 20 doctors and 13 nurses) were interviewed over a seven-month period. The results suggested that (i) the prospective assessment of post-traumatic amnesia was influenced by: knowledge; beliefs about consequences; environmental context and resources; skills; social/professional role and identity; and beliefs about capabilities; (ii) the use of guideline-developed criteria or decision rules to inform the appropriate use of a CT scan was influenced by: knowledge; beliefs about consequences; environmental context and resources; memory, attention and decision processes; beliefs about capabilities; social influences; skills and behavioral regulation; (iii) providing verbal and written patient information on discharge was influenced by: beliefs about consequences; environmental context and resources; memory, attention and decision processes; social/professional role and identity; and knowledge; (iv) the practice of providing brief, routine follow-up on discharge was influenced by: environmental context and resources; social/professional role and identity; knowledge; beliefs about consequences; and motivation and goals.

**Conclusions:**

Using the Theoretical Domains Framework, factors thought to influence the management of mild traumatic brain injury in the emergency department were identified. These factors present theoretically based targets for a future intervention.

## Background

Mild traumatic brain injury (mTBI) is a frequent cause of presentation to emergency departments (EDs), accounting for 80% of all head injury cases [1,2]. The majority of people with mTBI will make a full recovery within a couple of weeks or months [3]; however, a proportion (5% to 12%) will suffer from persistent symptoms that can lead to difficulties in returning to routine daily life such as work or school [4-6]. Of those who present to hospital, around 80% are discharged directly from the ED [7]. As the ED is often the only medical contact these people have, the care they receive has the potential to affect their outcome [8].

Several clinical practice guidelines (CPGs) have been developed to assist clinicians in managing mTBI. A study to identify and assess the quality of all CPGs for the management of mTBI in the ED found 18 CPGs and of these, 6 were identified as evidence-based and published in the last 10 years [9]. From these six CPGs, four key evidence-based recommended practices were identified (see Table 1) [9,10]. Despite the availability and wide dissemination of these CPGs, studies from the UK, Australia, Ireland, USA, Canada and Norway have found variability in how mTBI is managed [8,11-17]. A survey of ED Directors in Australia found that the majority of EDs did not use a validated tool to assess post-traumatic amnesia (PTA) in the ED [14]. Variations were found in the use of CT imaging [12,15], and studies from the USA found that only 51% of people with mTBI received written patient information [18] and that nearly 38% were discharged without recommendations for specific follow-up [8]. Studies have also reported variation in the content of the information leaflets provided, with little or no information on possible post-concussional symptoms [16,17].

**Table 1:** Key evidence-based recommended practices

Recommended practice 1	Post-traumatic amnesia should be prospectively assessed in the emergency department using a validated tool.
Recommended practice 2	Guideline-developed criteria or clinical decision rules should be used to determine the appropriate use and timing of CT imaging.
Recommended practice 3	Verbal and written information should be provided on discharge.
Recommended practice 4	Brief, routine follow-up consisting of advice, education and reassurance should be provided.

Many factors at different levels in the healthcare system can contribute to these variations in practice [19,20]. An understanding of these factors is needed to develop implementation interventions to increase the uptake of evidence into practice and so reduce variability in the delivery of these recommended practices. Such interventions are more likely to be effective if they target the factors influencing practice change compared to passive dissemination of CPGs or no intervention [21].

Using theories of behavior change to understand the factors influencing practice and design implementation interventions has the advantages of building on summaries of current knowledge and working within a framework that promotes the translation of empirical findings into new knowledge [22-24]. However, a systematic review of CPG implementation studies reported that only 22.5% had used theory to inform the design of the interventions and, of these, only 6% provided an explicit rationale for theory selection [25]. A method aimed to make available a wide range of theories relevant to behavior change for use in implementation research is the Theoretical Domains Framework (TDF) [26]. The TDF consists of 12 theoretical domains (groups of constructs from theories of behavior change) that can be considered when exploring influencing factors and designing interventions. The TDF has been validated to confirm the optimal domain structure, content and labels [27].

This study aimed to explore the factors that may influence the use of four key evidence-based recommended practices for managing mTBI in the ED (see Table 1), as perceived by ED clinicians. A secondary aim was to determine if there were differences in influencing factors with regard to location of hospital (metropolitan, regional) and professional group (medical, nursing). Findings from this paper will be used to develop a targeted, theory- and evidence-informed intervention to increase the uptake of evidence into practice and improve the management of mTBI in Australian EDs [28]. The development and evaluation of this intervention, as part of a cluster randomized controlled trial, will be reported separately.

## Methods

### Study design

This was a qualitative study using in-depth, semi-structured interviews.

### Participants

Participants were staff responsible for the clinical management of people with mTBI working in 24-hour hospital EDs within the Australian state of Victoria. These included medical doctors, registered nurses, nurse practitioners, and ED Directors. Recognizing that a hospital’s location and size could influence the hospital’s management practices for mTBI [29], we aimed to recruit a stratified purposive sample [30] of clinicians from a range of small to large metropolitan, inner and outer regional EDs to ensure all viewpoints were represented. The Australian Standard Geographical Classification-Remoteness Areas (ASGC-RA) [31] system was used to group hospitals in terms of remoteness (*i.e*., the physical distance of a location from the nearest urban centre). The aim was to interview at least one medical doctor per participating hospital and also the ED Director and a nurse. Sampling continued until saturation, with the stopping criteria being tested after each successive interview until there were three consecutive interviews without additional material [32].

### Procedure

Hospitals with a 24-hour ED were identified through a Government Health Information website. ED Directors received an invitation letter including explanatory statement and consent form. They were asked to indicate whether they would be willing to be interviewed and to forward copies of the documentation to relevant staff on behalf of the research team. Interview participants opted in to the study through completion of a consent form.

Single face-to-face interviews were conducted within their hospital at a time and location nominated by the participants. Two researchers (EJT, MB) conducted the interviews and took turns in leading the different topics discussed. This allowed the other researcher to concentrate on listening, asking clarifying questions, and thinking about the questions that needed further exploration. The researchers had experience in evidence-based medicine and qualitative research methods with knowledge of the clinical field and in-depth knowledge of the project.

Interviews were audiotaped and transcribed verbatim. Checked transcripts were imported into NVIVO 8 (QSR International Pty Ltd, Australia) to manage the data and facilitate the analysis. The date of the interview was added to the transcripts, allowing ‘tracking’ and development of the coding framework.

### Interview content

The interview guide consisted of two parts (see Additional file 1). The first part included broad questions about how people with mTBI were managed in their ED. The second part explored the four key evidence-based recommended practices in detail. Not all recommended practices were relevant to all clinicians (*e.g*., nurses don’t decide whether a patient needs a CT scan). However, their perceptions of the factors influencing their colleagues’ practice were explored. The interview guide was informed by the TDF [26] with questions formulated to explore each of the TDF domains. The interview guide was developed by investigators with expertise in behavior change and implementation research (ET, MB, DOC, SM, JF) and a practicing ED clinician (JK). The guide was piloted with two clinical staff.

### Analysis

Data were analyzed using an iterative process. Two researchers (ET/MB) independently reviewed the interview transcripts and open coded text relating to each of the recommended practices and the factors influencing those. These factors were coded to domains in the TDF [26]. When fragments were relevant to more than one domain, they were cross indexed. The researchers met after coding the first five interviews to discuss the coding. Discrepancies were discussed until consensus was reached. An audit trail was produced by keeping a record of coding decisions [33]. A domain was considered important according to saliency analysis (*i.e*., factors that were frequently mentioned, were deemed to be of high importance by the researchers or participants, or had both of these attributes) [34]. Quotations were used from the transcripts to illustrate each important domain [35]. Potential differences in influencing factors and domains with regard to professional group and location of hospital were explored.

### Ethics

Ethics approval was obtained from the Monash University Human Research Ethics Committee (MUHREC)–Project Number: CF10/2343–2010001338.

## Results

### Participants

Interviews were held over a seven-month period (November 2010 to May 2011). The interviews were predominantly held face-to-face; however, some were held by telephone due to the remote location of the hospitals. The interviews had a mean duration of 39 minutes (SD 9 minutes). Thematic saturation was reached after interviewing 42 participants (see Table 2 for characteristics of the participants).

**Table 2:** Characteristics of participants

**Hospital**	ASGC-RA^*^	**Size**^**#**^	**Director**	**Senior doctor**	**Doctor**	**Senior nurse**	**Nurse**	**Total**
1	Major Cities	Large	1	1				2
2	Major Cities	Large		1				1
3	Major Cities	Large		2				2
4	Major Cities	Large	1	3		1		5
5	Major Cities	Medium	1					1
6	Major Cities	Medium	1	3			1	5
7	Major Cities	Medium	1	2			1	4
8	Major Cities	Medium	1		1		1	3
9	Inner Regional	Large	1		4	1		6
10	Inner Regional	Medium					1	1
11	Inner Regional	Medium	1	2		2	1	6
12	Inner Regional	Small	1		1	1	1	4
13	Outer Regional	Small				1	1	2
		Total	9	14	6	6	7	42

^*^Australian Standard Geographical Classification–Remoteness Areas (RA1–major cities; RA2–Inner Regional; RA3–outer regional).

^#^Size according to annual ED patient presentations (<20,000 small; 21,000 to 49,000 medium; 50,000+ large).

Each of the recommended practices had its own pattern of influencing factors. Additional file 2: Tables S1 to S4 lists the factors perceived to influence practices, arranged by theoretical domain and clinician group. Illustrative quotations have been included, and although these were edited for readability, no substantive changes were made. Text that has been added for clarity has been placed in brackets. The following paragraphs summarize our findings.

### Post-traumatic amnesia should be prospectively assessed in the ED using a validated tool

#### Self-reported current practice

All doctors and nurses reported that they did not use a validated tool to prospectively assess for PTA, and they believed this is uncommon in ED practice in Australia. Most respondents reported using clinical questioning to assess whether patients were orientated and whether they had had retrograde or anterograde memory problems (*e.g*., asking what was the first and last thing they remember, before and after the event).

#### Factors influencing practice

The key factors thought to influence prospectively assessing for PTA in the ED using a validated tool were grouped within six theoretical domains (see Additional file 2: Table S1).

There was little knowledge about the availability of validated tools to assess PTA in the ED. Those who were aware of validated tools were unsure of the details or where to find the tools (‘I’m aware that [tools] exist. I don’t know the details of them, nor use them.’ ID 19.5, senior doctor).

Senior doctors reported that they felt comfortable determining whether a person with mTBI was in an amnesic state without the use of a validated tool. Some indicated that they did not see the additional benefit of using one, as they were not convinced that this would change the outcomes of their current management, *i.e*., change their decision to admit or discharge a patient (Beliefs about consequences). Several doctors and nurses thought that using a more objective measure of PTA could improve recording of patient care, but there were concerns that the use of a tool might be time consuming and increase the ED stay for these patients (Beliefs about consequences). (‘If I were to sit down and start 20 questions… that’s very time consuming and you could probably only do this once, when they’re in the short stay unit.’ ID 22.3, senior doctor).

Since none of the clinicians were using a validated tool to assess PTA, it was thought likely that skills and confidence in using a validated tool to assess PTA would need to be supported (Skills; Beliefs about capabilities). One senior doctor reported that many ED clinicians do not know how to assess for PTA, and several nurses attributed this to a lack of training (Skills). (‘I certainly haven’t been taught how to do an assessment [for PTA]. I have read about it but I’ve never done one. I was never shown how to do one.’ ID 6.3, nurse). There was some variation in the discussions around what constituted PTA and how it related to recommended practice.

With regard to environmental context and resources, both doctors and nurses stated that PTA guidance or tools were not available in their ED. Furthermore, observation charts currently in use do not have the space or contain prompts to include PTA information (‘We have the [Glasgow Coma Scale] section but we don’t have on our emergency department forms anywhere about amnesia…we don’t have anywhere where you can write specifically that actually prompts you to ask that question.’ ID 37.2, nurse).

With regard to social/professional role and identity, several doctors considered that using a validated tool to assess PTA was outside the role of the acute ED setting. (‘I think because it comes more from a rehabilitation end sort of area. The focus in emergency departments has always been on the CT… I think we’re caught up in looking for pathology…I think it’s been that it’s outside our scope of practice.’ ID 10.2, senior doctor). A team approach to managing mTBI in the ED was discussed particularly in undertaking and interpreting neurological observations (‘We have a team approach in the emergency department. Our nursing staff do neurological observations…it’s my responsibility to make sure I’m happy the patient’s not at risk and someone walking out in PTA has significant risk of making a poor judgement and ending up back in here with a more significant injury…I think it would be a medical and nursing role.’ ID 10.2, senior doctor).

There were few differences between the findings from regional-based and metropolitan-based clinicians. Clinicians from the regional hospitals were more likely to express the need for training in using a validated tool to assess PTA.

### Guideline developed criteria or clinical decision rules should be used to determine the appropriate use and timing of CT imaging

#### Self-reported current practice

All doctors stated that the decision to CT scan a patient is their responsibility and that they had 24-hour access to a CT-scanner. Doctors based in regional areas generally needed to call in a radiographer out of hours to access CT. Few doctors reported that they used clinical decision rules or CPGs to inform their decision-making process, and their hospitals rarely had CT scanning protocols for mTBI available. The majority of hospitals had supervision policies in place stating that the decision to CT (along with other imaging decisions) should be made in consultation with the most senior doctor on the floor. Overnight in regional or metropolitan hospitals, the availability of a senior doctor to support this decision-making process is limited. One regional hospital had developed and implemented a protocol based on guideline-developed criteria to assist junior doctors in gaining access to CT scans in times with less supervision.

#### Factors influencing practice

The key factors thought to influence the practice of using guideline-developed criteria or clinical decision rules to determine the appropriate use and timing of a CT scan were grouped within seven theoretical domains (see Additional file 2: Table S2).

Although most senior doctors were aware of CPGs or clinical decision tools to guide CT scanning decisions for mTBI, junior doctors were less likely to know about these (Knowledge). Senior doctors reported a preference to use their clinical experience rather than a decision rule to determine the need for CT. They considered the assessment of head injury as not always objective, and decision rules or criteria could not cover all options (Beliefs about consequences). (‘I know they’ve tried to make decision rules for CT heads. In injury they don’t work very well…it completely varies and there’s no hard or fast [rules].’ ID 19.4, senior doctor). Although the majority of doctors stated that they were aware of the radiation risks associated with CT scanning and the need to reduce a patient’s exposure to ionizing radiation, particularly if they are young, they articulated concerns about missing life-threatening events. Ordering a CT scan was seen as reassuring, confirming the patient was safe for discharge. (‘It takes one person that you miss and then it’s finger pointing asking why didn’t you CT scan when it is available? It’s very hard sometimes because if you’re in a culture where they do a lot of CT scanning, the consequences of not doing it, and there is a problem, is very high.’ ID 22.3, senior doctor).

Junior doctors found the decision more difficult due to their lack of experience and were more likely to want to scan these patients (Beliefs about capabilities). (‘The hard and fast rules are great when you are learning but you’ve got to use a mix of that and your experience as well I think.’ ID 4.2, senior doctor).

The majority of doctors stated that their hospital had policies in place for junior doctors to discuss their CT scanning decision with senior members of staff. This was particularly important in regional hospitals with a high number of junior or less experienced doctors (Behavioral regulation).

A consistent finding across all interviews was the environmental context and resources of the ED and the pressure on ED staff to discharge patients quickly due to increasing ED presentations and workload. When an ED is nearing full capacity, ordering a CT scan was seen as a quicker way of discharging the patient safely. (‘People are colored by situation.. if the place is absolutely going off and you know you are going to struggle to go back in and check on that person and there are two junior nurses out there… the risk benefit for the greater good is just to scan the brain and make sure we are not missing something…our practice is impacted by the moment.’ ID: 10.1, senior doctor). The increasing availability of the CT scan was seen as a key factor influencing its increased use and the reduced need to be selective about its use. (‘The CT scan unfortunately has become like a chest x-ray. It’s become almost like a screening tool.’ ID 22.3, senior doctor).

Several doctors who were aware of clinical decision rules for mTBI thought they were complicated and difficult to remember (Memory, attention and decision processes*)*. (‘I know they’ve tried to make decision rules for CT heads… some are too complicated to apply anyways, because there’s too many criteria.’ ID 19.4, senior doctor).

With regard to social influences, several doctors indicated that there was a changing culture in Australia to scan most adult patients with mTBI rather than observe. (‘There is a changing pattern going on here. I was going to say the more experienced but maybe the older medical staff won’t scan everyone with a period of loss of consciousness. The more junior staff will scan everybody who’s had a loss of consciousness.’ ID 25.1, senior doctor). Radiologists were infrequently consulted in the decision-making process to CT scan adults with mTBI, and it was suggested that different professional groups have different CT thresholds. Some ED doctors felt there was a pressure on them from in-patient consultants to scan the majority of patients with mTBI before admission, and specialist registrars who had been trained at trauma centres were more likely to want to scan more often.

The biggest differences between regional and metropolitan hospitals were in environmental contextual factors. Regional hospitals were less likely to have 24-hour access to a radiographer out-of-hours and therefore have to be selective about which patients receive a CT scan. These hospitals were also more likely to have a greater proportion of junior staff. In light of their circumstances, they were more likely to suggest the use of decision rules to inform the decision to CT.

### Verbal and written patient information should be provided on discharge

#### Self-reported current practice

Doctors and nurses stated that they try to provide verbal and written information to people with mTBI on discharge; however, there are circumstances when they are likely to forget. Some hospitals had the information in printed format, and others had it on the intranet for the clinician to print out. One hospital provided a DVD in addition to a printed information sheet. A variety of patient information sheets from a range of sources were used. Clinicians often used the one they remember or can access on the internet or intranet. Some doctors may include additional information or revise the sheet according to what information they want to provide to a particular patient.

#### Factors influencing practice

The key factors thought to influence the practice of providing verbal and written patient information on discharge were grouped within five theoretical domains (see Additional file 2: Table S3).

The majority of clinicians interviewed thought it was important to provide verbal and written patient information to people with mTBI. The beneficial beliefs about consequences of providing this information included: providing patients with the information to identify key deterioration signs so that they return to the ED (‘If they’re being discharged they need some education in regards to their head injury…what signs to look out for in case there’s complications as a result of the head injury and they should present to the hospital rather than delaying it.’ ID 4.3, nurse); reducing the risk of litigation for the doctor if these symptoms are missed; and reducing the patient’s anxiety by providing information on what symptoms they can expect after receiving a head injury and prevent unnecessary re-presentations to the ED. (‘The most critical function of all is that people don’t worry about a symptom that they’ve got two or three days down the track and come back…certainly adequate information at the time of discharge prevents representation.’ ID 24.5, doctor). Written patient information was seen of particular importance as patients were often unlikely to remember what they were told in the ED. It was felt that junior staff may be less aware of the consequences of not providing this information due to their lack of experience and the lack of education on the importance of providing the information (Knowledge).

With regard to environmental context and resources, a wide range of electronic and paper-based patient information sheets, of varying quality, were used. Several doctors and nurses stated that their current patient information sheets were out of date and difficult to find on the Intranet. (‘It looks quite old the stuff we give out and we’re going through a process of updating stuff and as soon as I see something that’s got 2002 written on it, I am not using it…you feel much better giving them out when you know it’s up to date information.’ ID 25.3, senior nurse). Several reported that the stock of printed patient information sheets often ran out and was not replaced. The pressure on ED staff due to the increasing numbers of ED presentations and the use of casual and pool staff in the ED were also stated as contributing factors to patients not receiving patient information. With a high turnover of medical staff, it was difficult for the current staff to keep new staff trained and informed about where the patient information is held and the importance of providing this information. (‘In most EDs there’s a large flow of casual and pool staff who don’t get orientation. So they don’t know that [the patient information] is there. The ANUMs [Associate Nurse Unit Managers] are under such pressure they don’t get time to remind every nurse.’ ID 25.2, senior nurse).

The majority of doctors and nurses interviewed stated that they sometimes forget to provide written patient information on discharge (Memory, attention and decision processes). Several reasons were provided, and these largely related to the ED environment, *e.g*. lack of time, busyness of the ED, rather than by intention. (‘Look definitely I forget about it, we’re all humans. Most likely if I am busy with another patient.’ ID 24.2, doctor).

With regard to social/professional role and identity, all of the doctors felt that it was their role to provide patient information, as they are responsible for the decision to discharge a patient. There was, however, a range of responses regarding the role of the nurses. Some of the doctors felt that it was a joint responsibility and that nurses were skilled and more vigilant about providing written information to the patient. Some doctors did not see it as the role of the nurse. Although the nurses agreed that it was the role of the doctor to decide on whether the patient was safe for discharge, several felt that the provision of patient information was a shared role as it crosses into patient advocacy and ensuring that the patient is fully informed. A shared role was emphasized as important in hospitals with a high turnover of medical staff who might not know where the patient information sheet was.

There were no differences between the findings from regional-based and metropolitan-based clinicians.

### Brief, routine follow-up consisting of advice, education and reassurance should be provided

#### Self-reported current practice

All but one of the hospitals included in this study had a policy of referring people with mTBI to their general practitioner (GP) on discharge from the ED or providing them with advice to see a GP if they continue to have problems. Patients were either discharged with a letter to their GP, or the letter was faxed directly to their GP. One hospital had a policy of routinely referring people with mTBI to an acquired brain injury (ABI) clinic if they satisfied the criteria for a CT scan. Clinicians based at another hospital, that had previously formed links with an ABI service, were unsure if this service was still available. In both cases, the ABI clinic had contacted the ED directly to request that they refer people with mTBI to them.

#### Factors influencing practice

Key factors thought to influence providing brief, routine follow-up consisting of advice, education and reassurance were grouped within five theoretical domains (see Additional file 2: Table S4).

All of the clinicians stated that they would be unable to provide routine follow-up to people with mTBI due to the increasing ED workload and current staffing levels and resources (Environmental context and resources). (‘We see too many patients a day to even consider it. Once they leave here well that’s it, the next lot comes through. We have too many things to worry about.’ ID 25.4, senior nurse). Organizing a referral for these patients to a specialist ABI service was seen as difficult, particularly for hospitals without a neuro-rehabilitation service or those located in regional area.

Following up people with mTBI to identify potential long-term problems was not seen as the role of the ED (Social/professional role and identity). Doctors reported the role of the ED as dealing with acute problems/emergencies and then identifying the most appropriate place to refer them for follow-up. This is largely done by referring them to their GP.

Although the majority of clinicians were aware that some people with mTBI go on to have long-term problems, they were unsure of the extent of the problem and did not have enough information on the link between severity of the head injury and the need for follow-up (Knowledge). Several doctors and nurses stated that they did not have information on the services available for people with mTBI or how to organize a referral. (‘I don’t think I’ve ever specifically organized neuropsych review. I don’t know how to do it in this place. I am not aware specifically of that sort of head injury follow-up. I’m not aware of any specific services.’ ID 19.4, senior doctor).

Referring people with mTBI to their GP rather than organizing a follow-up was seen as favorable to the ED by reducing the number of re-presentations and the workload and pressure on ED staff (Beliefs about consequences). Several doctors expressed concern that some people might not follow up with their GPs due to long waiting times or not being able to afford to see them. On average, the out-of-pocket expense for a GP consultation in Australian is $26.97 [36]. Some worried about potentially flooding an ABI clinic with minor cases if they referred all people with mTBI to them.

There was a low motivation (Motivation and goals) by doctors to identify those that might develop long-term complications due to the perceived lack of follow-up services available, other than GPs, particularly in regional areas. (‘If there was some way of following up or there was some way of actually helping that patient in a meaningful way other than the information you can give them there at the time, then I think that would be quite motivating to find these people and look a bit harder for them …when there’s nothing to do for them, then there’s zero motivation.’ ID 25.6, doctor).

Clinicians from both regional and metropolitan hospitals felt that the ED was under-resourced and not the appropriate place to follow-up these patients. Both groups were unsure of the prevalence of long-term problems for this patient group and when a follow-up was needed. Although both groups were unsure of the services available for referring a person for follow-up from the ED, regional hospitals without a neuro-rehabilitation service found this particularly difficult.

## Discussion

This study used the TDF to explore the factors influencing the management of mTBI in the ED. To our knowledge, no other study of this nature has been published to date on this issue. Three domains were identified as being consistently important for all four recommended practices: knowledge; beliefs about consequences; and environmental context and resources.

For all four recommended practices, some clinicians were unaware of tools or services available to manage mTBI (Knowledge). This was particularly true for screening tools to assess PTA. One of the factors contributing to this may be the fact the ED deals with a wide range of conditions, and this poses challenges in terms of keeping up to date with tools and guidance available. With regard to beliefs about consequences, beliefs were predominantly positive in favor of three of the four recommended practices in terms of the effect on patients (assessing PTA; provision of verbal and written discharge information; and provision of brief, routine follow-up), although doctors expressed concerns regarding the use of a validated tool to assess for PTA, as it would not change their subsequent management decisions. Finally, the environmental context and resources domain was coded frequently as having an influence on all recommended practices. Consistently, the workload pressures on the ED were mentioned. This is in line with the results of other studies that have explored the factors influencing the ED management of other clinical conditions [37-39]. For example, Bessen conducted interviews with ED and radiology staff to explore the factors influencing the uptake of the Ottawa ankle rule in the ED [39]. The staffing of the ED (*i.e*., high turnover and shift work) and the workload pressures on the ED (*e.g*., ordering of radiographs at busy times in the ED to improve the flow of patients) were discussed. This correlates with the findings in this paper; when workload in the ED is high, ordering a CT scan without the use of guideline criteria or clinical decision rules was stated as a way to quickly discharge a person and free up a bed. Environmental context and resources was also the domain where we identified differences between metropolitan and regional hospitals. For example, participants at regional hospitals were more likely to express difficulties in relation to staffing and available resources (*e.g*., radiology at night) and express the need for guideline-developed criteria or clinical decision rules in times of reduced supervision and reduced access to CT.

Social/professional role and identity was deemed as important for three of the recommended practices (assessing PTA; provision of verbal and written discharge information; and provision of brief, routine follow-up). This domain was considered important in instances where participants reported that there was a lack of clarity regarding the person responsible for the recommended practice, or where the practice was not perceived as feasible in the ED setting.

The results of this paper will inform a theory- and evidence-informed intervention to increase the uptake of the recommended practices. The process of using the TDF to explore factors influencing the uptake of evidence into practice and then using this information to guide the choice of intervention components has been previously used by some of the author team to design a complex intervention to improve acute low back pain management in primary care [24]. We will adopt this general approach to intervention design and apply it in a new (secondary care) setting. When designing interventions aiming to bring about practice change in organizational settings, it is important to recognize that adoption of a particular intervention can take place at multiple levels (*e.g*., organizational and individual). Therefore, a companion paper provides additional analysis of the broader organizational context of the ED environment in relation to change. It draws upon organizational theory to explore how organizational factors influence the organization and delivery of mTBI care and practice change in the ED. By tailoring our implementation intervention to the results described in these two papers, we hope to maximize the effectiveness of our implementation intervention.

Although this study has some clear strengths (such as using a theoretical framework to explore influencing factors; recruitment continuing until there was a saturation of themes using a rigorous method [32] in over 40 participants; and two researchers undertaking the interviews, independently coding and analyzing the results), there are some potential limitations.

Firstly, as this is a qualitative study, the identified influencing factors are the views of the clinicians interviewed and therefore do not provide evidence of the actual influences on practice [40]. The factors influencing practice are also identified in a snapshot of time, so they may change over time once practice change starts to occur. For example, when the interviews were undertaken, the clinicians were not using a validated tool to assess PTA; therefore, these interviews only assess the influencing factors from clinicians not performing this practice. Ideally, we would have sampled both groups of people (*e.g*., those using a validated tool and not using a validated tool to assess PTA) for all practices to identify any salient differences in factors influencing those practices.

## Conclusion

Using the TDF, factors thought to influence the management of mTBI in the ED were identified. Each of the four recommended practices had its own pattern of influencing factors. These factors present theoretically-based targets for a future intervention.

## Abbreviations

CPG: Clinical Practice Guideline; ED: Emergency Department; mTBI: mild Traumatic Brain Injury; TDF: Theoretical Domains Framework; PTA: Post-Traumatic Amnesia.

## Competing interests

Denise O’Connor and Susan Michie are Associate Editors for Implementation Science. All decisions on this manuscript were made by another editor. All other authors declare that they have no competing interests.

## Authors’ contributions

EJT/MB carried out the interviews and analyzed the results. EJT drafted the manuscript. SG/RG/DOC conceived the study. All authors revised the manuscript for important intellectual content and gave final approval of the version to be published.

## Supplementary Material

Additional file 1 Interview guide for Emergency Department (ED) clinical staff. Click here for file

Additional file 2: Table S1 Factors thought to influence practice to prospectively assess PTA in the ED using a validated tool. **Table S2.** Factors thought to influence the practice of using guideline developed criteria or clinical decision rules to assess for high or low risk of intracranial injury to determine the appropriate use and timing of CT imaging. **Table S3.** Factors thought to influence the practice of providing verbal and written information on discharge. **Table S4.** Factors thought to influence the practice of providing brief, routine follow-up consisting of advice, education and reassurance. Click here for file
