# Supplementary material for: Understanding practice: the factors that influence management of mild traumatic brain injury in the emergency department-a qualitative study using the Theoretical Domains Framework
Source: Implement Sci. 2014 Jan 13;9:8. doi: 10.1186/1748-5908-9-8 (PMC3895840; doi:10.1186/1748-5908-9-8)
Supplement: Additional file 1 — Interview guide for Emergency Department (ED) clinical staff. [file 1748-5908-9-8-S1.docx]

**Additional file 1: Interview guide for Emergency Department (ED) clinical staff**

**Introduction**

As outlined in the explanatory statement, this study aims to identify and explore the factors that influence the management of patients with mild traumatic brain injury.

You do not have to answer every question and can cease the interview at any time. If you need to attend to an urgent matter we can stop the interview and recommence it later.

We will talk about how you define mild traumatic brain injury (mTBI), how patients with mTBI are managed in the ED setting and the factors that influence this management. We are interviewing ED staff to learn from their experiences, including many departments to get a broad view.

Before we start do you have any questions?

**Background information**Do you use the term mild traumatic brain injury (mTBI), if so, how would you define it?

Approximately, how many patients are seen in this ED in a week/month with mTBI?

What are the key demographic characteristics of these patients e.g. sex, age?

**Management of mild traumatic brain injury (mTBI) – brief overview of care pathway steps**Can you talk me briefly through the various steps in how patients with mTBI are managed in your ED?

This is just to get an idea about which ED health professionals are involved in the management of patients with mTBI and, briefly, what do they each do? We will talk about some of the steps of the care pathway in more detail later.

So, firstly, what happens when a patient with suspected mTBI first presents to the ED department?

- Who is responsible for assessing the severity of the brain injury on presentation at the ED?
  - What happens once they are assessed, what happens next?
- Do you have a CT scanner available at the hospital?
  - If no, what happens if a patient with mTBI needs to have a CT scan?
  - Prompts: Use of x-ray? Extended observation? Referral?

Could we now discuss some of the different steps in the care pathway in more detail?

**Recommended practice: post-traumatic amnesia should be prospectively assessed in the ED using a validated tool**Understanding the nature of the behavior

So, firstly we are interested in the initial clinical assessment of patients with (suspected) mTBI

- What does the clinical assessment of patients with mTBI involve?
  - Prompts: Which tests? Does it include GCS and/or PTA? How soon after presentation are the tests conducted?)
- Who conducts the assessment(s)/test(s)?
- Where are the results of the assessments/tests recorded? (and by who?)
- How are the results used? (and by who?)
- How do you [or others, as relevant] determine whether a patient is at high or low risk of brain injury?
- Are there any particular clinical signs or symptoms that you [or others, as relevant] would consider as high risk? How would you measure these?
- Are there any other tests or tools that are used in the assessment of these patients that we haven’t covered? (if so, what are they, who conducts them, when, how are they recorded, how are they used?)
- Do you undertake a PTA assessment?
  - If yes, do you use a validated tool?
  - If no, have you noticed if PTA is an issue for these patients?
  - How do you pick up on it?
- Are there any particular pathways for this type of patient?

Prompt questions to explore factors influencing practice (grouped by TDF domains).

| **TDF Domains** | **TDF Definitions [Constructs][25]** | **Prompt questions** |
| --- | --- | --- |
| Knowledge | An awareness of the existence of something. [Knowledge including knowledge of condition/scientific rationale. Procedural knowledge. Knowledge of task environment.] | Are you familiar with risk assessment tools e.g. GCS/PTA tools?  Do you know how to interpret results to distinguish between high and low risk? |
| Skills | An ability or proficiency acquired through practice. [Skills Skills development Competence Ability Interpersonal skills Practice Skill assessment] | Do you know how to do it/able to use tools?  What skills are needed? |
| Social professional role and identity | A coherent set of behaviors and displayed personal qualities of an individual in a social or work setting. [Professional identity Professional role Social identity Identity Professional boundaries Professional confidence Group identity Leadership Organizational commitment] | Do you think conducting risk assessments e.g. measurement of PTA is part of your role? Do you think interpreting these risk assessment results and determining the patient’s risk is an appropriate part of your role? |
| Beliefs about capabilities | Acceptance of the truth, reality, or validity about an ability, talent, or facility that a person can put to constructive use. [Self-confidence Perceived competence Self-efficacy Perceived behavioral control Beliefs Self-esteem Empowerment Professional confidence] | Any difficulties in assessing risk (e.g. measuring PTA using validated tools)?  Any challenges in determining risk in general and using different tests/tools in particular? |
| Beliefs about consequences | Acceptance of the truth, reality, or validity about outcomes of a behaviour in a given situation. [Beliefs Outcome expectancies Characteristics of outcome expectancies Anticipated regret Consequences] | Benefits / disadvantages of determining high /low risk and doing GCS/PTA?  Consequences of doing or not doing? |
| Motivation and goals | A conscious decision to perform a behavior or resolve to act in a certain way. Mental representations of outcomes or end states that an individual wants to achieve. [Stability of intentions Stages of change model Transtheoretical model and stages of change Goals (distal/proximal) Goal priority Goal/target setting Goals (autonomous/controlled) Action planning Implementation intention] | Why do you do these tests?  Are there incentives to do them?  Do you feel you have to? |
| Memory, attention and decision processes | The ability to retain information, focus selectively on aspects of the environment and choose between two or more alternatives. [Memory Attention Attention control Decision making Cognitive overload/tiredness] | Would you ever forget to do any of the relevant tests?  Is it something you do routinely? |
| Environmental context and resources | Any circumstance of a person’s situation or environment that discourages or encourages the development of skills and abilities, independence, social competence, and adaptive behavior. [Environmental stressors Resources/material resources Organizational culture/climate Salient events/critical incidents Person x environment interaction Barriers and facilitators] | Do resources influence whether you assess these patients (e.g. for PTA using validated tool)?  Are there tools available?  Are there sufficient human resources?  Are there clear communication channels?  Are there sufficient physical resources?  Do you have enough time/do you have competing demands?  Does the working environment of the ED have an effect? |
| Social influences | Those interpersonal processes that can cause individuals to change their thoughts, feelings, or behaviors. [Social pressure Social norms Group conformity Social comparisons Group norms Social support Power Intergroup conflict Alienation Group identity Modeling] | Do you seek opinions of colleagues in risk assessment / interpreting test results?  What are the views of your colleagues?  Do you observe others e.g. have role models? |
| Emotion | A complex reaction pattern, involving experiential, behavioral, and psychological elements, by which an individual attempts to deal with a personally significant matter or event. [Fear  Anxiety Affect Stress Depression Positive/negative effect Burn-out] | Is this behavior difficult to deal with?  Would you prefer to avoid this behavior? |
| Behavioral regulation | Anything aimed at managing or changing objectively observed or measured actions. [Self-monitoring Breaking habit Action planning] | What would you need to do to undertake an assessment?  Are there any protocols to facilitate the assessment? |

**Recommended practice: guideline-developed criteria or clinical decision rules are used to determine the appropriate use/timing of CT imaging**
Understanding the nature of the behavior

- Would all patients with mTBI be referred for CT scan?
  - If not, under what circumstances would they not be referred for CT?
- Who would make the decision to refer or not refer a patient for a CT scan? And how is the decision made? E.g. are you able to decide yourself, or do you need to consult with others/need approval from others?
- Would you consult with the radiologist in the decision? Or other colleagues?
- Would you involve the patient in the decision? If so, how?
- What is the process for referring a person with mTBI for a CT scan? Once you have referred a patient for a CT scan, how long should it take before the patient is scanned?
- If a patient with mTBI is referred for CT, does that mean that the patient will always get a CT? Are there situations in which someone or something prevents the actual CT?
- Where is the referral for CT recorded?
- Who interprets (and/or reviews) the results of the CT scan and where are these results recorded?
- Who acts on the results of the CT scan? What typically happens?

If no CT scan at site:

- If the patient is deemed at higher risk for complications, what happens next?
- Who would take the decision to transfer / admit patient for observation?

Prompt questions to explore factors influencing practice (grouped by TDF domains).

| **TDF Domains** | **TDF Definitions [Constructs][25]** | **Prompt questions** |
| --- | --- | --- |
| Knowledge | An awareness of the existence of something. [Knowledge including knowledge of condition/scientific rationale. Procedural knowledge. Knowledge of task environment.] | Are you familiar with decision rules to determine the intracranial risk for these patients? |
| Skills | An ability or proficiency acquired through practice. [Skills Skills development Competence Ability Interpersonal skills Practice Skill assessment] | Do you know how to assess the intracranial risk for these patients? |
| Social professional role and identity | A coherent set of behaviors and displayed personal qualities of an individual in a social or work setting. [Professional identity Professional role Social identity Identity Professional boundaries Professional confidence Group identity Leadership Organizational commitment] | Do you think referring a patient for imaging is part of your role? Do you think using decision rules to inform your decision to CT is part of your role? Do you think interpreting results of a CT scan is an appropriate part of your role? |
| Beliefs about capabilities | Acceptance of the truth, reality, or validity about an ability, talent, or facility that a person can put to constructive use. [Self-confidence Perceived competence Self-efficacy Perceived behavioral control Beliefs Self-esteem Empowerment Professional confidence] | Do you have any difficulties in referring for CT? Do you have any difficulties using decision rules to inform your decision to CT?  Are there any challenges in negotiating with patients?  Are there any challenges in negotiating with radiologist? |
| Beliefs about consequences | Acceptance of the truth, reality, or validity about outcomes of a behavior in a given situation. [Beliefs Outcome expectancies Characteristics of outcome expectancies Anticipated regret Consequences] | What are the benefits / disadvantages of CT scan?  What are the benefits/disadvantages of using decision rules to inform your decision to CT?  Are there consequences of doing or not doing CT scan?  Are there any consequences of using decision rules to inform your decision to CT? |
| Motivation and goals | A conscious decision to perform a behavior or resolve to act in a certain way. Mental representations of outcomes or end states that an individual wants to achieve. [Stability of intentions Stages of change model Transtheoretical model and stages of change Goals (distal/proximal) Goal priority Goal/target setting Goals (autonomous/controlled) Action planning Implementation intention] | Why do you CT scan?  Do you routinely refer patients for a CT? Do you routinely use a decision rule to inform your decision to CT?  Do you feel you have to?  Are there any incentives to refer a patient? |
| Memory, attention and decision processes | The ability to retain information, focus selectively on aspects of the environment and choose between two or more alternatives. [Memory Attention Attention control Decision making Cognitive overload/tiredness] | Is this something you do routinely? |
| Environmental context and resources | Any circumstance of a person’s situation or environment that discourages or encourages the development of skills and abilities, independence, social competence, and adaptive behavior. [Environmental stressors Resources/material resources Organizational culture/climate Salient events/critical incidents Person x environment interaction Barriers and facilitators] | Do resources influence whether you scan a patient?  Are there CT decision rules available?  Are there sufficient human resources?  Are there clear communication channels?  Are there sufficient physical resources?  Do you have enough time/do you have competing demands?  Does the working environment of the ED have an effect? |
| Social influences | Those interpersonal processes that can cause individuals to change their thoughts, feelings, or behaviors. [Social pressure Social norms Group conformity Social comparisons Group norms Social support Power Intergroup conflict Alienation Group identity Modelling] | Do you seek opinions of colleagues in CT scanning decisions?  What are the views of your colleagues on CT scanning? What are the views of your colleagues on using decision rules to inform CT decision making?  What are the patient views on CT scanning?  What would your colleagues do?  What are the views of management?  Do you observe others e.g. have role models?  Is there shared decision making? |
| Emotion | A complex reaction pattern, involving experiential, behavioral, and psychological elements, by which individual attempts to deal with a personally significant matter or event. [Fear  Anxiety Affect Stress Depression Positive/negative effect Burn-out] | Is this behavior difficult to deal with? |
| Behavioral regulation | Anything aimed at managing or changing objectively observed or measured actions. [Self-monitoring Breaking habit Action planning] | Are there any protocols/guidance available to guide this behavior? What would you need to assess intracranial risk in this patient group? |

**Recommended Practice: verbal and written information should be provided on discharge**Understanding the nature of the behavior

- What is the process for discharging people with mTBI from the ED? Who is involved?
- Do all patients with mTBI receive patient discharge information? What type of information do they receive? (content, format, mode of delivery etc)
- If we asked the last 10 patients with mTBI seen in this ED, how many would say they had received discharge information?
- Where can the discharge information be found in the ED?
- If it is provided, is the provision of discharge information recorded? If so, where is it recorded? Who would do this? Is it clear who’s responsibility this is?
- Can I take a copy of the discharge information you typically provide to patients with mTBI?

Prompt questions to explore factors influencing practice (grouped by TDF domains).

| **TDF Domains** | **TDF Definitions [Constructs][25]** | **Prompt questions** |
| --- | --- | --- |
| Knowledge | An awareness of the existence of something. [Knowledge including knowledge of condition/scientific rationale. Procedural knowledge. Knowledge of task environment.] | Familiar with content of patient discharge information?  Familiar with existing brochures / leaflets? |
| Skills | An ability or proficiency acquired through practice. [Skills Skills development Competence Ability Interpersonal skills Practice Skill assessment] | Do you know how to explain this information to the patient?  What skills are needed? |
| Social professional role and identity | A coherent set of behaviors and displayed personal qualities of an individual in a social or work setting. [Professional identity Professional role Social identity Identity Professional boundaries Professional confidence Group identity Leadership Organizational commitment] | Do you think providing patient information is part of your role? |
| Beliefs about capabilities | Acceptance of the truth, reality, or validity about an ability, talent, or facility that a person can put to constructive use. [Self-confidence Perceived competence Self-efficacy Perceived behavioral control Beliefs Self-esteem Empowerment Professional confidence] | Any there any difficulties in doing it?  Are there any challenges in providing info?  Is it easy/difficult? |
| Beliefs about consequences | Acceptance of the truth, reality, or validity about outcomes of a behavior in a given situation. [Beliefs Outcome expectancies Characteristics of outcome expectancies Anticipated regret Consequences] | Are there benefits / advantages of providing information?  What are the consequences of not doing it? |
| Motivation and goals | A conscious decision to perform a behavior or resolve to act in a certain way. Mental representations of outcomes or end states that an individual wants to achieve. [Stability of intentions Stages of change model Transtheoretical model and stages of change Goals (distal/proximal) Goal priority Goal/target setting Goals (autonomous/controlled) Action planning Implementation intention] | Why would you provide patient discharge information?  Do you routinely provide patient discharge information?  Do you feel you have to?  Are there any incentives to provide this information? |
| Memory, attention and decision processes | The ability to retain information, focus selectively on aspects of the environment and choose between two or more alternatives. [Memory Attention Attention control Decision making Cognitive overload/tiredness] | Would you ever forget to provide information?  Is it something you do routinely? |
| Environmental context and resources | Any circumstance of a person’s situation or environment that discourages or encourages the development of skills and abilities, independence, social competence, and adaptive behavior. [Environmental stressors Resources/material resources Organizational culture/climate Salient events/critical incidents Person x environment interaction Barriers and facilitators | Do resources influence whether you provide info?  Are there any tools e.g. brochures / leaflets available Are there sufficient human resources?  Is there clarity in the team roles? Are there clear communication channels?  Are there sufficient physical resources?  Do you have enough time/do you have competing demands?  Does the working environment of the ED have an effect? |
| Social influences | Those interpersonal processes that can cause individuals to change their thoughts, feelings, or behaviors. [Social pressure Social norms Group conformity Social comparisons Group norms Social support Power Intergroup conflict Alienation Group identity Modelling] | What are the views of your colleagues regarding the importance of providing information?  Do you observe others e.g. have role models? |
| Emotion | A complex reaction pattern, involving experiential, behavioral, and psychological elements, by which individual attempts to deal with a personally significant matter or event. [Fear  Anxiety Affect Stress Depression Positive/negative effect Burn-out] | Is this behavior difficult to deal with?  Would you prefer to avoid this behavior? |
| Behavioral regulation | Anything aimed at managing or changing objectively observed or measured actions. [Self-monitoring Breaking habit Action planning] | What would you need to do to provide discharge info?  Are there any protocols available?  What would help? |

**Recommended Practice: brief, routine follow-up consisting of advice, education and reassurance on discharge from the ED should be provided.**Understanding the nature of the behavior

- Following discharge does the ED have any further contact with these patients?
  - Do you have any contact with the patient’s GP?
  - Do you refer the patient to their GP?
  - Do you sometimes refer these patients to other services e.g. allied health?
  - Do you have any contact with allied health services regarding these patients?

Prompt questions to explore factors influencing practice (grouped by TDF domains).

| **TDF Domains** | **TDF Definitions [Constructs][25]** | **Prompt questions** |
| --- | --- | --- |
| Knowledge | An awareness of the existence of something. [Knowledge including knowledge of condition/scientific rationale. Procedural knowledge. Knowledge of task environment.] | Familiar with services or processes of how to organize follow-up for patients with mTBI? |
| Skills | An ability or proficiency acquired through practice. [Skills Skills development Competence Ability Interpersonal skills Practice Skill assessment] | Do you know how to organize a follow-up for this patient?  What skills are needed? |
| Social professional role and identity | A coherent set of behaviors and displayed personal qualities of an individual in a social or work setting. [Professional identity Professional role Social identity Identity Professional boundaries Professional confidence Group identity Leadership Organizational commitment] | Do you think following-up a mTBI patient is part of your role? |
| Beliefs about capabilities | Acceptance of the truth, reality, or validity about an ability, talent, or facility that a person can put to constructive use. [Self-confidence Perceived competence Self-efficacy Perceived behavioral control Beliefs Self-esteem Empowerment Professional confidence] | Any there any difficulties in doing it?  Are there any challenges in organizing a follow-up?  Is it easy/difficult? |
| Beliefs about consequences | Acceptance of the truth, reality, or validity about outcomes of a behavior in a given situation. [Beliefs Outcome expectancies Characteristics of outcome expectancies Anticipated regret Consequences] | Are there benefits / advantages of following up mTBI patients?  What are the consequences of not doing it? |
| Motivation and goals | A conscious decision to perform a behavior or resolve to act in a certain way. Mental representations of outcomes or end states that an individual wants to achieve. [Stability of intentions Stages of change model Transtheoretical model and stages of change Goals (distal/proximal) Goal priority Goal/target setting Goals (autonomous/controlled) Action planning Implementation intention] | Why would you organize follow-up?  Do you routinely follow-up mTBI patients?  Do you feel you have to?  Are there any incentives to do this? |
| Memory, attention and decision processes | The ability to retain information, focus selectively on aspects of the environment and choose between two or more alternatives. [Memory Attention Attention control Decision making Cognitive overload/tiredness] | Would you ever forget to organize follow-up for this patient?  Is it something you do routinely? |
| Environmental context and resources | Any circumstance of a person’s situation or environment that discourages or encourages the development of skills and abilities, independence, social competence, and adaptive behavior. [Environmental stressors Resources/material resources Organizational culture/climate Salient events/critical incidents Person x environment interaction Barriers and facilitators] | Do resources influence whether you organize follow-up?  Are there sufficient human resources?  Is there clarity in the team roles? Are there clear communication channels?  Are there sufficient physical resources?  Do you have enough time/do you have competing demands?  Does the working environment of the ED have an effect? |
| Social influences | Those interpersonal processes that can cause individuals to change their thoughts, feelings, or behaviors. [Social pressure Social norms Group conformity Social comparisons Group norms Social support Power Intergroup conflict Alienation Group identity Modelling] | What are the views of your colleagues regarding the importance of following up these patients?  Do you observe others e.g. have role models? |
| Emotion | A complex reaction pattern, involving experiential, behavioral, and psychological elements, by which individual attempts to deal with a personally significant matter or event. [Fear  Anxiety Affect Stress Depression Positive/negative effect Burn-out] | Is this behavior difficult to deal with?  Would you prefer to avoid this behavior? |
| Behavioral regulation | Anything aimed at managing or changing objectively observed or measured actions. [Self-monitoring Breaking habit Action planning] | What would you need to do to organize follow-up?  Are there any protocols available?  What would help? |

If time permits and only if these items have not yet been covered:

- What do you think are the key actions/decisions when managing a patient with mTBI so that it would maximize the beneficial outcomes for the patients?
- Is there an aspect of the patient pathway we should pay more attention to in future interviews?
- If there was one thing you could change in your hospital to improve the management of mTBI in ED what would you change?
- Is there anything else about the assessment of patients with suspected brain injury that you would like to mention that is not already covered?
- Do you have any additional comments on the content of the interview or feedback on how the interview went?

**THANK YOU VERY MUCH FOR YOUR TIME**
