# Supplementary material for: Understanding practice: the factors that influence management of mild traumatic brain injury in the emergency department-a qualitative study using the Theoretical Domains Framework
Source: Implement Sci. 2014 Jan 13;9:8. doi: 10.1186/1748-5908-9-8 (PMC3895840; doi:10.1186/1748-5908-9-8)
Supplement: Additional file 2: Table S1 — Factors thought to influence practice to prospectively assess PTA in the ED using a validated tool. Table S2. Factors thought to influence the practice of using guideline developed criteria or clinical decision rules to assess for high or low risk of intracranial injury to determine the appropriate use and timing of CT imaging. Table S3. Factors thought to influence the practice of providing verbal and written information on discharge. Table S4. Factors thought to influence the practice of providing brief, routine follow-up consisting of advice, education and reassurance. [file 1748-5908-9-8-S2.docx]

**Additional file 2 (Table S1 to S4):**

Table S1: Factors thought to influence practice to prospectively assess PTA in the ED using a validated tool

| **Key Domain** | **Themes** | **Dr** | **Nurse** | **Representative quotations** |
| --- | --- | --- | --- | --- |
| **Knowledge** | Limited knowledge of what PTA is or how to assess it.        Limited knowledge of validated tools to assess PTA in the ED.          Unaware of guidelines that specify the length of PTA that would indicate the need for further investigation.  Aware that PTA is an indication of brain injury. | ✓          ✓            ✓      ✓ | ✓          ✓                  ✓ | “[PTA] is something a lot of emergency consultants don’t know about. A lot of our registrars don’t know. We had a talk from one of our rehab specialists who mentioned PTA several times and I had to actually say “wait a minute” and looking at the faces of the registrars...I think because it comes more from a rehabilitation end sort of area [they are not aware]. The focus in emergency departments has always been on the CT...I think we’re very caught up in looking for pathology. If there’s no contusion, if there’s no particular haemorrhage, if there’s no small subarachnoid bleed then we call the CT normal, when the patient is clearly not…I think it’s been that it’s something outside our scope of practice.” (ID 10.2: SD, RA1, M)  “I’m aware that [formal PTA assessment tools] exist. I don’t know the details of them, nor use them.” (ID 19.5: SD, RA1, L)  “I don’t know of any tools [to assess PTA in the ED].” (ID 10.1: SD, RA1, M)  “No [I am not aware of any formal PTA assessment tools]…when you mentioned that I thought that could probably be quite handy…if it gives us any better indicators of care or the level of head injury it would certainly be helpful.” (ID 38.2: N, RA2, M)  I’m sure there’s probably a [amnesia] tool out there that might make it a whole lot easier but not something that I use in my day to day practice.” (ID 24.5: D, RA2, L)  “I suppose that the determinant for us would be the duration of PTA but we don’t have any specific guideline for whether 10 minutes or an hour or a day is considered a trigger for any further investigation.” (ID 19.5: SD, RA1, L)  “It is part of one of the things that make you worry if they do have post-traumatic amnesia then you worry more about the severity of the head injury.” (ID 39.3: D, RA2, S) |
| **Key domain** | **Themes** | **Dr** | **Nurse** | **Representative quotations** |
| **Environment context and resources** | No mandated validated tool to assess PTA available in the ED.       No space on ED forms to include amnesia/PTA information. Amnesia/PTA assessment is not included in clinical pathway  Using a tool to assess PTA could only be done once in the short stay unit.  Staff in the ED work in teams therefore the PTA tool needs to facilitate communication between staff members and there needs to be a person responsible for driving the data collection.  Many clinical decision tools are used in the ED. To be used they need to change clinical management, take little time and have inter-user reliability. | ✓                 ✓     ✓         ✓ | ✓ | “There is no structured [PTA] tool. So there is no mandated tool. There is some information about head injury in the guideline section of the ED intranet, I don’t know that everyone refers to it on every patient and is done I suppose more as an FYI or if some resident is wanting more information. Because our level of supervision is relatively high, a lot of that is usually done by directly escalating it to the consultant.” (ID 3.1: D, RA1, L)  “We don’t have a formalised [PTA] assessment tool.” (ID 19.5: SD, RA1, L)  “We don’t actually have anywhere on our emergency department forms anywhere about amnesia…we don’t actually have anywhere where you can write specifically that actually prompts you to ask that question...I can’t remember if amnesic state is actually on the pathway, I think it is more about vital signs and their GCS and everything. I don’t think the amnesic state actually is on there.” (ID 37.2: N, RA3, S)    “If I were to sit down and start 20 questions with the thing, that’s very time consuming and you could probably only do this once, when they’re in the short stay unit.” (ID 22.3: SD, RA1, M)   “I think it’s the time that is the issue and if it’s useful in communication between professionals. So if one staff member performs the rule...and then indicates the findings to another staff member, does that mean anything in terms of what might happen for that patient.” (ID 19.5: SD, RA1, L)  “[It’s] purely time pressures... if you have a scoring system or a sheet or proforma to fill out, who owns it? Who’s driving that data collection? Who’s making sure it’s done efficiently and effectively and that the patients agree to it?” (ID 22.4: SD, RA1, M)  “We have a lot of clinical decision tools that come into use in the Emergency Department and may or may not achieve sustained use, for a large variety of conditions. And time is a factor, inter-user reliability is a factor, and decision-making influence is a factor. So if the tool doesn’t change our management it may not achieve widespread use.” (ID 19.5: SD, RA1, L) |
| **Key Domain** | **Themes** | **Dr** | **Nurse** | **Representative quotations** |
| Skills | Limited skills and training in assessing patients for neurological conditions.   Limited training on how to assess PTA using validated tools. |  | ✓     ✓ | “We’re not very good with neuro stuff here because we don’t have a neuro service so we don’t have neurologists, it’s basically what we learn along with everything else when you do an emergency course.” (ID 25.3: SN, RA2, M)   “I’m not aware of any [validated tools] and it is very difficult [to assess PTA] because we’ve never really had any training on it.” (ID 37.2: N, RA3, S)  “I certainly haven’t been taught how to do an assessment [for PTA]. I have read about it but I’ve never done one. I was never shown how to do one.” (ID 6.3: N, RA1, M) |
| **Key domain** | **Themes** | **Dr** | **Nurse** | **Representative quotations** |
| **Beliefs about consequences** | Using a tool to assess PTA is more time consuming than using clinical questions and experience.  Using a validated tool to diagnose PTA and potential long-term outcomes is not seen as useful to the acute ED setting.  Do not see the additional benefit of using a validated tool to assess PTA rather than using clinical experience.  Do not believe that assessing patients for amnesia, including PTA, is a concern for the ED as they are not involved in the follow-up of these patients.  Do not know if there are any benefits to using a validated tool to assess for PTA.  Using a validated tool to assess PTA improves documentation of care.  Using a validated tool to assess PTA is more sensitive and reproducible than informal screening.  A consequence of diagnosing a patient with amnesia or PTA is that it indicates they may have a brain injury and advice should be modified to include information on avoiding a second injury (PTA assessment guides subsequent treatment decisions)  Would worry about the severity of a patient’s head injury if PTA is diagnosed.  Consequences of not diagnosing a patient with amnesia or PTA are that you could miss a brain injury and they don’t get follow-up. | ✓  ✓          ✓  ✓         ✓      ✓  ✓     ✓ | ✓       ✓  ✓ | “If I were to sit down and start 20 questions with the thing, that’s very time consuming and you could probably only do this once, when they’re in the short stay unit.” (ID 22.3: SD, RA1, M)  “I think it’s the time that is the issue and if it’s useful in communication between professionals. So if one staff member performs the rule...and then indicates the findings to another staff member, does that mean anything in terms of what might happen for that patient.” (ID 19.5: SD, RA1, L)  “[the difficulty] purely time pressures and if you have a scoring system or a sheet or proforma, who owns it, who’s driving that data collection, who’s making sure it’s done efficiently and effectively and that the patients agree to it?” (ID 22.4: SD, RA1, M)  “ ...picking up of PTA and whether it’s important for long-term outcomes. It’s not important in the acute setting. If this is a really important…a critical thing then we’ll do it, but we have to be given a good reason, ‘cause it’s not useful for us.” (ID 4.3: N, RA1, L)    “I’m usually comfortable with my [amnesia] assessment personally. I mean obviously that comes from years of clinical experience...as opposed to maybe a junior doctor who doesn’t have that degree of clinical experience.” (ID 19.3: SD, RA1, L)  “So I think you pick up [amnesia] on clinical clues. There’s nothing formal to do that. So I would probably find it easier as a very experienced clinician as opposed to a junior...hopefully at least by the process of triage, even if a junior nurse came in and was working it up, it would be already flagged because a senior ED nurse would have already gone it is a proper head injury.” (ID 22.5: SN, RA1, M)  “I don’t think that amnesia is a particular concern, we don’t see them later on so I don’t know what they’re like later. That’s the problem with emergency you don’t know what actually happens further down the track, we don’t get follow-up.” (ID 22.2: SD, RA1, M)  “I don’t know whether them trying to do a formal assessment picks out things because I’ve never done it and I don’t know whether there is any benefit in doing it.” (ID 22.3: SD, RA1, M)   “To have something you could document [PTA] on would be really good. I think it’d be helpful for any issues they have later on down the track too.” (ID 25.3: SN, RA2, M)   “At the end of the day, my informal screening for most patients is not as good and reproducible as a Westmead. If the nurse has done a Westmead: tick, tick, tick there you go it’s in the history and 2 years later you can say well there it was…there’s distinct advantages for having those sort of tools and they’re probably more sensitive.” (ID 25.6: SD, RA2, M)  “Amnesia or PTA has a correlation with brain injury. Subtle neuropsychiatic deficits like amnesia probably point to some sort of pathologic process that occurred during the trauma even if it’s not detectable on normal standard imaging...may have a prognostic significance in terms of avoidance of a second hit injury. The advice given to the patient in terms of specific activities to avoid would be modified based upon the PTA assessment.” (ID 19.5: SD, RA1, L)        “It is part of one of the things that make you worry if they do have post-traumatic amnesia then you worry more about the severity of the head injury.” (ID 39.3: D, RA2, S)   “I think you have to identify that they’ve had some traumatic brain injury, that there has been some evidence of amnesia or loss of cognition and I think that process of identifying is crucial. Because if you don’t ask the correct questions or make the correct enquiries at the initial presentation you’ll probably miss it. And the subsequent follow-up of the cognition or the thoughts of the patient or how they were feeling can be missed if you’re not following it up. If there is no-one to follow it up they may fall through the cracks.” (ID 22.4: SD, RA1, M) |
| **Key domain** | **Themes** | **Dr** | **Nurse** | **Representative quotations** |
| **Social/professional role and identity** | Assessing for PTA is seen as outside the role of the ED.   Unsure of who is responsible for completing and promoting the validated PTA tool.  Assessing for PTA identifies long term problems therefore it has limited value or role in the acute ED setting.      Assessing patients for PTA was seen as both the role of the doctor and the nurse. | ✓     ✓               ✓ | ✓ | I think because it comes more from a rehabilitation end sort of area [ED are not aware]. The focus in emergency departments has always been on the CT...I think we’re very caught up in looking for pathology...I think it’s been that it’s something outside our scope of practice.” (ID 10.2: SD, RA1, M)  “[It’s] purely time pressures...if you have a scoring system or a sheet or proforma to fill out, who owns it? Who’s driving that data collection? Who’s making sure it’s done efficiently and effectively and that the patients agree to it?” (ID 22.4: SD, RA1, M)    “Prior to introducing it, to what value it’s going to have for the patient’s care? Because you’re in the emergency department, you’re looking at the short term, you know, what’s happening there? I think it’s more obviously the long term.” (ID 39.4: SN, RA2, S)  “ ...picking up of PTA and whether it’s important for long-term outcomes. It’s not important in the acute setting. If this is a really important…a critical thing then we’ll do it, but we have to be given a good reason, ‘cause it’s not useful for us.” (ID 4.3: N, RA1, L)  “We have a team approach in emergency. Our nursing staff do neurological observations and often have a very good feel in that regards…it’s my responsibility to make sure I’m happy the patient’s not at risk and someone walking out in PTA has significant risk of making a poor judgment and ending up back in here with a more significant injury…I think it would be a medical and nursing role.” (ID 10.2: SD, RA1, M) |
| **Key domain** | **Themes** | **Dr** | **Nurse** | **Representative quotations** |
| Beliefs about capabilities | Do not find amnesia assessment difficult and know what to look for.  Others find it difficult and there is inconsistency in their assessments.  Junior doctors find it more difficult due to their limited clinical experience.   Find it difficult to assess for anterograde amnesia as more subjective.    Find it difficult to assess amnesia in patients with dementia. | ✓     ✓     ✓ | ✓ | “I don’t find it particularly hard myself [amnesia assessment], I sort of know that’s what I need to look out for. I know other people do and then another nurse will come on and notice that they’re having some amnesia and it’s like “why wasn’t this picked up earlier”? It’s because not everyone’s consistent in their assessments.” (ID 25.2: SN, RA2, M)    “I’m usually comfortable with my [amnesia] assessment personally. I mean obviously that comes from years of clinical experience...as opposed to maybe a junior doctor who doesn’t have that degree of clinical experience.” (ID 19.3: SD, RA1, L)    “I think retrograde amnesia, most of the patients will have it, but the anterograde it’s difficult to assess. If it’s difficult I always go to the safest option and do a CT. Sometimes the patient really couldn’t give a good history...I think it’s a bit more subjective.” (ID 24.2: D, RA2, L)  “...it’s usually quite straightforward in the straightforward patient; but if it’s someone with dementia or something everything is difficult.” (ID 4.4: SD, RA1, L) |
| **Key domain** | **Themes** | **Dr** | **Nurse** | **Representative quotations** |
| Motivation and goals # | Not motivated to assess PTA in the ED as not convinced it is important for the acute setting.   PTA should be assessed in the ED and recorded in the notes. | ✓ | ✓ | “If picking up of PTA and whether it’s important for long-term outcomes. It’s not important in the acute setting. If this is a really important…a critical thing then we’ll do it, but we have to be given a good reason, ‘cause it’s not useful for us.” (ID 4.4: SD, RA1, L)  “I think it’s definitely something we should be doing. It’s something we should be recording and it should be recorded when they start getting some recollection as well.” (ID 25.3: SN, RA2, M) |

# Domain deemed as relevant but not a ‘key’ domain.

Table S2: Factors thought to influence the practice of using guideline developed criteria or clinical decision rules to assess for high or low risk of intracranial injury to determine the appropriate use and timing of CT imaging

| **Key domain** | **Themes** | **Dr** | Nurse (*) | **Representative quotations** |  |
| --- | --- | --- | --- | --- | --- |
| **Beliefs about consequences of using CT head scanning criteria or rules** | Not convinced head scanning rules are useful.       .         The consequence of using a head scanning rule is that you end up scanning everyone who is old and there are a few things that are not practical.  The consequence of using the American guidelines is a tendency to scan too early or too quickly.  Head scanning rules are useful when you are learning.  Head scanning rules are a safety net for junior staff. | ✓                  ✓        ✓        ✓    ✓ |  | “I know that they’ve tried to make decision instruments for CT heads. In injury and they don’t work very well...it completely varies and there’s no hard or fast – or some of them are too complicated to apply anyways, ‘cause there’s too many criteria.” (ID 19.4: SD, RA1, L)   “There’s some established rules like the Canadian Head Rule, I’m not convinced that they are overly useful. I suppose I’ve developed something I feel works for me... I feel that what I do works. The thing is with head injury...it may not be something particularly objective, you just think this person doesn’t look right and I don’t think you can write a policy for that.” (ID 10.1: SD, RA1, M)  “The evidence base probably points towards the fact when a clinical problem is complex then decision rules don’t have the same clinical usefulness as probably the application of clinical experience. Or if it’s an ethereal subject about the clinical decision making based upon everything from education to findings of a bedside to local trends in practice. The one size fits all problem of applying a fairly rigid set of determinants to something can be quite nuanced. I think that’s where the attempts to derive a decision rule have struggled.” (ID 19.5: SD, RA1, L)  “They’re not too bad [head scanning rules], the problem is that you end up scanning everyone who is old. There are a few things that aren’t on a practical level that I just ignore for those rules. So you end up being selective about which rule I use...with Canadian I would have to scan them, NEXUS suits me this time.” (ID 25.6: SD, RA2, M)      “I’m often concerned about some of the American guidelines, because I think they’re driven much more than our practice or British practice by medico legal concerns. I think defensive medicine, we’re more intelligent than that, we can practice better than that. We’re always going to miss things but I think when you are driven by medical and legal concerns then the patient’s not the focus of the practice. I think there’s been a tendency in a lot of the American literature to scan too early or too quickly whereas the UK guidelines and I think Canada is better.” (ID 10.2: SD, RA1, M)  “...the hard and fast rules are great when you’re learning but you’ve got to use a mix of that and your experience as well I think.” (ID 4.2: SD, RA1, L)   “I use the rules that I’ve written as my day to day practice and I tell the junior medical staff that they should know these rules, they should be able to recite them or be able to find them so that they can follow them in practice because it’s their safety net.” (ID 37.2: D, RA2, S) |  |
| **Beliefs about consequences of CT scanning** | Consequences of not CT scanning a patient are missing a life threatening event and inappropriately discharging an impaired patient.              Ordering a CT scan is less expensive than observing a patient for 4 hours.  CT brains are more acceptable than CT scans of the chest or abdomen due to the amount of radiation.  Ordering a CT scan is easy and you can discharge a patient.  Ordering a CT scan in a rural or regional hospital involves organising a transfer by ambulance to another hospital, reducing the availability of the ambulance for the hospital. Aware that there are associated radiation risks with a CT scan.     The risk of radiation to the patient influences the decision to order a CT. | ✓                    ✓     ✓      ✓               ✓       ✓ | ✓ | “Well there’s the possibility that you’re going to miss a life threatening event.” (ID 22.2: SD, RA1, M)   “It’d look bad if you get someone with significant injury and you miss it. And so because those stories exist and around and everyone is quite aware of it. You can’t help put an element of defensivity at times in your practice.” (ID 19.4: SD, RA1, L)  “The likelihood of scanning someone’s head very much relates to the last case they were involved in or the last miss...you know a colleague missed a small extradural last year and for the next 3 months you’re going to be wanting to scan everyone. So the reality is that as much as those tools are available at the end of the day we’re responsible for the clinical decision and protocols are just there to guide is...we are coloured by recent experience.” (ID 10.2: SD, RA1, M)  “In a metropolitan or tertiary hospital with the availability of radiology...you would be more inclined to do a CT. Because it takes one person that you miss and then its finger pointing asking why didn’t you CT scan when it is available? It’s very hard sometimes because if you’re in a culture where they do a lot of CT scanning, the consequences of not doing it, and there is a problem, is very high.” (ID 22.3: SD, RA1, M)  “Well there’s implications of timing and finance, 4 hours in a bed compared to a CT it’s probably more expensive to spend 4 hours in a bed.” (ID: 22.2: SD, RA1, M)     “CT brains are more like chest x-rays and they’re more acceptable compared to if you did a CAT scan of the chest or abdomen, it’s about the amount of radiation.” (ID 22.3: SD, RA1, M)    “The culture in Australia is if there’s any little hiccup in any way I am going to scan you, anything because it’s easy because then I’ve got a normal scan and I can send you home... and that radiation stuff no-one thinks about.” (ID 25.1: D, RA2, M)  “It might actually influence the fact that we might transfer a patient [after hours] that we otherwise would have kept, if we really felt it needed to be done...we’d have to organise an ambulance and after hours there’s one ambulance for our area. So if it went to Melbourne, there’d be no ambulance down here...and obviously we’re sending a patient away from their home and they’ve got to get home.” (ID 39.1: N, RA2, S)      “I think there is enough evidence to say that there is a burden of medical radiation...we had a teaching day and one of the topics was medical radiation, it is quite current at the moment, so quite a few people talk about it.” (ID 25.6: SD, RA2, M)  I think over the years, the threshold has lowered dramatically despite the apparent known risk of radiation.” (ID 19.4: SD, RA1, L)  “There is a significant trend or environmental change perhaps towards markedly reducing patients’ exposure to ionising radiation. One of the factors that influence decision making at the moment is the desire to minimise radiation exposure in all patients but specifically or more importantly perhaps in the patients with a large number of years of potential cumulative dose exposure.” (ID 19.5: SD, RA1, L) |  |
| **Key domain** | **Themes** | **Dr** | Nurse (*) | **Representative quotations** |  |
| **Environment context and resources** | The ED is busy and the workload high. It is easier to CT scan a patient and discharge them to free up the bed.  The decision to CT scan is dependent on the ED environment. In stressful times the risk benefit for the greater good is to scan the head.  Ordering a CT scan is easier than in the past.  Overnight and when the ED is busy it is easier to scan.  CT head scanning protocols are not available.   Reduced access to radiology services in rural areas at the week-end and out of hours.                  CT scanning protocols and guidelines are available on the Intranet and in hard copy. | ✓        ✓   ✓        ✓                       ✓ | ✓                      ✓    ✓ | “I think it’s the case we’re so busy and our workload is so high...I think they just figure “look it’s easier, we’ll CT everyone and then we’ll sort it out later...there’s a waiting room full of patients and we need those beds.” (ID 25.2: SN, RA2, M)     “People would be coloured by situation and I think it’s important to be open about this. If the place is absolutely going off and you know you are going to struggle to go back in and check on that person and there’s two junior nurses out there, then I might say “look in this situation, the risk benefit for the greater good is just to scan the brain and make sure we are not missing something because we won’t pick up the subtle things as quick as we otherwise might in the ideal world”. I think you need to acknowledge that we are, our practice is impacted by the moment.” (ID 10.1: SD, RA1, M)  “...the CAT scan unfortunately has become, like, a chest x-ray. It’s become almost like a screening tool.” (ID 22.3: SD, RA1, M)   “Overnight and when it’s busy the potential oversight scrutiny can potentially be less. It’s easier to do a scan.” (ID 4.1: D, RA1, L)   “I don’t even think we have a protocol you know, who to CT and who not to CT. Basically I think it comes down to the senior consultant, they’re the ones who have to okay it.” (ID 25.4: SN, RA2, M)  “We have a radiographer who starts at 7 and they work through until 11, and then we’ve got an on call after 11...we call them in if we need them and he’s normally only called in for emergencies.” (ID 25.2: SN, RA2, M)  “There’s gaps, so night shift is a particularly high risk, when there’s no consultants. From midnight to 8am there’s a registrar on and CT is less accessible. They feel they have to get the radiographer out of bed...so they would feel less likely, less inclined to call a radiographer to get a report.” (ID 25.6: SD, RA2, M)  “Every now and then we have issues with the radiologists on call overnight, when [they] actually don’t do CT brains, they tend to either just do radiology or ultrasound...situation where we have had to send patients to [name] for CT brains which is 2 and a half hours down the road...After hours is an issue we often have radiographers to do the CT brains [the results are] then sent offshore, and you can be waiting up to four to five hours to actually get results back...you just hope you have a doctor on that can read CT brains.” (ID 37.2: N, RA3, S)  “If [city based hospital] are waiting on a CT brain report [out of hours] to accept a patient from the country you know you have a 3 to 4 hour wait to get the CT report and then sometimes another 4 hours for transport, especially if ambulances overnight might not be available. So some patients are with us for 8-10 hours before they can be in specialist care.” (ID 37.2: SN, RA3, S)  “...we’ve got a reasonably sturdy CT or imaging guideline for closed head injury, particularly in the minor head injury group which we term 14 to 15, ...our guideline involves who to scan, who not to scan, who to observe, who not to observe.” (ID 6.2: D, RA2, M) |  |
| **Key domain** | **Themes** | **Dr** | Nurse (*) | **Representative quotations** |  |
| **Memory, attention and decision processes** | Clinical decision rules are complicated and difficult to remember | ✓ |  | “I know they’ve tried to make decision rules for CT heads…some are too complicated to apply anyways, ‘cause there’s too many criteria.” (ID 19.4: SD, RA1, L)   “I think they’re good [Canadian head rule] but the problem is they are hard to remember. You know I can never remember, they’re complicated you know. I mean it’s simple but to remember all the ins and outs...two or three vomits and I think there’s something about 65 years and over what about 63 and 364 days?” (ID 19.3: SD, RA1, L) | |
| **Key domain** | **Themes** | **Dr** | Nurse (*) | **Representative quotations** |  |
| **Beliefs about capabilities** | Junior doctors find the decision to CT scan difficult as they do not have the clinical experience.     Rural doctors sometimes find the decision to CT difficult due to reduced accessibility to CT and limited supervision   Difficult to assess the need for a CT scan if the patient has alcohol on board.   It is not difficult to make the decision to order a CT scan if it is done with consultation.  Find it fairly clear to determine if someone has an injury and needs a CT scan. | ✓        ✓  ✓      ✓      ✓ |  | “The younger staff do not have the clinical experience. They’re worried they’re going to miss something they better do a CT, and order a CT or they’ll be told off by someone for not doing one.” (ID 19.3: SD, RA1, L)  “This is one of the things that I find tricky and when you’re working with lots of different consultants, which will be my next, sort of go-to point…to scan or not to scan their head.” (ID 24.4: D, RA2, L)  “It’s quite tricky sometimes because we don’t always have the option of doing a CT scan or asking a senior doctor to come and have a look with us.” (ID 39.3: D, RA2, S)  “One thing I found a bit confusing and end up doing more and more CTs are the patients who have alcohol on board and come in with head injuries...it’s always stressful to see that the patient has alcohol involved and whether to put it as a mild head injury or get alarmed and just get a CT brain. It gets fuzzy with the alcohol to determine how serious they are.” (ID 24.3: D, RA2, L)  “No it’s not that hard. I mean it’s as I say always done with consultation.” (ID 25.5: SD, RA2, M)     “I try to be fairly black and white and I think it’s usually fairly clear whether someone’s got an injury.” (ID 25.6: SD, RA2, M) |  |
| **Key domain** | **Themes** | **Dr** | Nurse (*) | **Representative quotations** |  |
| **Social influences** | Observing a patient with mTBI is seen as old school and not the norm.    The decision to CT scan is doctor dependent.  Junior staff are more likely to scan patients.          CT is being used more like a screening tool  ED doctors have a different scanning threshold to other professional groups.  Staff from trauma centres are more likely to scan patients.           Radiology is rarely involved in the decision-making process to order a CT scan for an adult patient with mTBI.      Radiologists are willing to do a CT head scan for patients with mTBI without consultation to senior staff and therefore the barriers have been removed.   In-patient consultants require a CT scan before admitting a patient with mTBI and disregard the ED doctor’s decision.  Patients with mTBI sometimes have expectations to receive a CT scan.     ED is a heavily supervised environment.  Junior doctors need to ask the supervising ED consultant when ordering a CT scan.  Interns need have sign off from a registrar to order a CT scan. | ✓       ✓    ✓          ✓   ✓     ✓  ✓          ✓  ✓  ✓        ✓    ✓ | ✓ | “Some people tend still to just do the 4 hour observation rule which is kind of old school but some people still do it. I tend to get a CT. The radiology department a number of years ago complained about the number of normal CT scans that we had. I think the head of the department had a quick chat with them...they looked at a normal CT scan as a failure of clinical decision making whereas we said that’s probably not how we look at it.” (ID 22.2: SD, RA1, M)  [The decision to CT scan] is “clinician dependent and perhaps people do use guidelines such as the Canadian CT head rule but I’ve not seen it widely applied in the ED and people tend to use clinical experience.” (ID 19.5: SD, RA1, L)  “There is a changing pattern going on here. I was going to say the more experienced but maybe the older medical staff won’t scan everyone with a period of loss of consciousness. The more junior staff will scan everybody who’s had a loss of consciousness.” (ID 25.1: SD, RA2, L).  “...some junior staff have rotated from other hospitals where they do CT on just about everything without any sort of thought process... some of the patients they're seeing are much more high acuity, but there's got to some sort of thought ...to think about who, do they really need it.” (ID 19.3: SD, RA1, L)  “The CAT scan unfortunately has become, like, a chest x-ray. It’s become almost like a screening tool.” (ID 22.3: SD, RA1, M)   “[Scanning rates] are varied between different groups of professionals. So emergency physicians would have a different threshold from radiologists, from neurosurgeons, from general surgeons.” (ID 19.5: SD, RA1, L)   “I think some of the junior doctors don’t ask first and some of the supervision at times falls down when the place is busy. There is a tendency for some of the surgical registrars who’ve been at trauma centres to want to scan lots of things and that sort of rubs off down the line from them.” (ID 22.1: SD, RA2, M)  “I hear from people that come here, that there is a much lower threshold for brain, for doing scans in the trauma centres. Just the scanner is there, they are easier to get, you don’t want to miss something. And we, rightly or wrongly, think that we are a bit more subtle about it than that, whether that’s a true perception of reality, you know? I mean, the other thing is, because, probably we overall see a potentially milder end of the spectrum, we spend a lot more time trying not to scan, whereas it may be that it’s…I don’t know. That’s the perception. I don’t know whether that’s the reality or not.” (ID 10.1: SD, RA1, M)  “Well at the end of the day, they're not the person looking at the patient and I don't want them to be obstructive which we have had, not as much now, but in previous, we have had some obstructive Radiology Registrars. I mean their Consultants are usually not an issue...most of the time it's usually our decision. Sometimes there might be discussion about the type of, not whether imaging was needed, it's more what would be the appropriate imaging modality, obviously head trauma's CT, but for other sort of trauma related or non-trauma related things we might use their opinion.” (ID 19.3: SD, RA1, VL)  “Radiology is only involved in decisions about children.” (ID 25.5: SD, RA2, M)  “For a CT brain radiology are happy to do them and so because they don’t have to ask a radiology consultant they just have to give it to a technician. Although the party line would be that the junior doctor should ask the consultant on the floor to get it, does that happen? No. Not a lot as the barrier’s been removed, if you haven’t got an ugly person to deal with in another department you don’t need to bother challenging and having a debate with your consultant who may disagree so you take the easy path.” (ID 25.1: SD, RA2, M)  “...in-patient unit Consultants...it's easier [CT scan] if they don't want to be responsible for missing anything, maybe it's also laziness on their behalf, not coming in and actually looking at the patient and teaching their junior staff...what do I as an Emergency Specialist know, I know nothing to some of these units...here's some disrespect to Emergency Physicians shown by some of the in-patient staff...is quite upsetting at times.” (ID 19.3: SD, RA1, L)    “...there’s often a patient expectation as well. So that’s another thing that has to be managed and that varies. Some people you can have that discussion and say, “Look, I really think it’s unlikely, you know, CT scan, provides radiation. I don’t think it’s going to add anything.” And some people are happy and other people just want the scan. So, you know, depending upon how senior and confident you are and, whether you could be bothered going through the whole process with a patient particularly if they’ve got a demanding parent or partner or – or whatever. That – that I’m sure can be a factor.” (ID 4.1: D, RA2, L)  “We have a fairly heavily supervised environment, for a less supervised environment then guidelines are good.” (ID 25.6: SD, RA2, M)   “Generally speaking, junior doctors who needed to ask would be directed by the supervising ED consultant.” (ID 10.1: SD, RA1, M)    “...the registrar actually has to sign off on, you know, the fact that they’re having a CT. The intern can’t just go and order one.” (ID 37.2: SN, RA3, S) |  |
| **Key domain** | **Themes** | **Dr** | Nurse (*) | **Representative quotations** |  |
| **Knowledge** | Not aware of a CT scanning guideline or tool. Decision to CT for mTBI is not part of education programme.   Do not know which consultant to ask about whether to scan the head.  Aware of head injury guidelines.  Aware of Canadian head injury rules. | ✓      ✓  ✓   ✓ |  | “No, I mean there may well be [a guideline or tool] but to be honest I haven’t actually gone around and done a whole lot of reading around it or anything. It’s not something that comes up through our education programme or anything like that. If there is such a thing I’m certainly not aware of it and I don’t use it.” (ID 24.4: D, RA2, L)   “This is one of the things that I find tricky and when you’re working with lots of different consultants, which will be my next, sort of go-to point to scan or not to scan their head.” (ID 24.4: D, RA2, L)   “I’m using the NICE guidelines because I come from UK.” (ID 24.2: D, RA2, L)   “I know about the Canadian head injury rules and I look and refer to them sometimes but the hard and fast rules are great when you are learning and you’ve got to use a mix of that and your experience as well.” (ID 4.2: SD, RA1, L) |  |
| **Key domain** | **Themes** | **Dr** | Nurse (*) | **Representative quotations** |  |
| **Behavioural regulation** | Junior staff cannot request a CT scan without consultation with a senior member of staff.   Imaging guideline provides a safety net for junior staff and provides them with the ability request a scan out of hours. | ✓       ✓ | ✓ | “The registrar actually has to sign off on the fact that they’re having a CT. The intern can’t just go and order one.” (ID 37.2: N, RA3, S)  “Junior medical staff can’t request a CT even directly from radiology without the consultant being involved or overnight registrar in charge. So they have to go through a senior member of staff to anything like that done.” (ID 6.2: SD, RA1, M)  “It was a time where we had difficulty with access to imaging out of hours and so we felt that we needed a guideline that gave the junior medical staff the ability to ring radiology and say we need a scan...we don’t have as much trouble accessing imaging but we still prefer that people stuck to a guideline because it gives you that institutional sort of support..it is their safety net.” (ID 24.5: D, RA2, L)  “So the intranet just sits in the background and its quite easy, and in fact, you know the radiologists complained a couple of months ago because someone ordered stuff just that, because it’s on the guideline.” (ID 3.2: SD, RA1, L) |  |

(*) The target clinician group for this behaviour is doctors however the impressions from nurses about what they think are the key factors influencing the behaviour of the doctors have been included.

Table S3: Factors thought to influence the practice of providing verbal and written information on discharge

| **Key domain** | **Themes** | **Dr** | **Nurse** | **Representative quotations** |
| --- | --- | --- | --- | --- |
| **Beliefs about consequences** | Unsure of the consequences and usefulness of providing written information as unsure how many people read it.  Junior staff are unaware of the consequences of not providing information.  Believe the majority of patients with mTBI will not have any complications.  A consequence of not providing information is that the patient deteriorates and does not call an ambulance or represent to the ED.  The motivation for the clinician to provide patient information is the fear of a patient deteriorating and not representing to the ED.    A consequence of providing information is that it gives the patient a better understanding of what’s normal.  Providing written information to the patient, reminds them of the verbal information they were told in the ED, in case they might have forgotten.  Providing patient information reduces the anxiety of the patient and prevents representation.     Not providing patient information is bad patient care and can affect the outcome of the head injury. | ✓            ✓            ✓        ✓  ✓  ✓         ✓ | ✓          ✓       ✓                    ✓                       ✓ | “I don’t know what the consequences actually would be, because I mean if you’re providing the information and they’re not reading it, then that’s the same as effectively not giving them it other than for the purpose of intent you’ve actually tried a little bit more.” (ID 24.4: D, RA2, L)  “Junior staff not so much...we try and encourage it but I suppose they’re not aware of the consequences that can happen really...but I suppose until they’ve kind of experienced a patient quite unwell I don’t think that they’d probably get it.” (ID 37.2: N, RA3, S)   “Most of them are not going to have anything serious, you know development of a complication later on but I suppose there’s potential that they may not recognise signs that there could be something developing or they should return to the hospital. I think for our patients...I say probably 98-99% will have no consequences.” (ID 19.3: SD, RA1, L)  “Definitely important and we should provide it more than what we do. They need to know what to look for as well as consequences, things that could happen further down the track. They might deteriorate during the night and they if they don’t know what to look for...then they are not going to know to call an ambulance or represent to the ED.” (ID 25.2: SN, RA2, M)   “The focus is to make sure that they come back and get help if things get worse. There is very little information [in the patient information] on what to expect and getting on with their life. That’s not the motivation...it is the fear that something might happen or they might bleed and that you want them to come back when that happens, so you don’t have to go and explain to the coroner. So that’s the fear and motivation. Does it actually help patients? Probably not...other than making them feel like they’ve got some information which patients generally feel better about.” (ID 25.6: SD, RA2, M)  “If they’re being discharged they need some education in regards to their head injury…what signs to look out for in case there’s complications as a result of the head injury and they should present to the hospital rather than delaying it.” (ID 4.3: N, RA1, L)  “We very, very rarely have patients coming back with complications from a minor head injury so from that point of view it’s probably not that important. I think that is why I like the [name] one because it gives information about what they can expect about their concentration and their headaches, what’s going to happen in the next few weeks and how to treat themselves. It’s not really for the acute big deterioration that might happen. It’s really just for the patients to give them a better understanding what’s normal and education about what’s normal.” (ID 4.4: D, RA1, L)   “It’s essential. I think written information is well supported as a central part of the Emergency Department discharge process because of the fact that people either are too stressed to remember what they’re told or like most of us can forget things they’re told. Or have further questions that they meant to ask but didn’t ask at the time which may be answered in the written documentation.” (ID 19.5: SD, RA1, L)  “Patients themselves might not take a lot of it in and not even that they have a head injury, patients don’t take a lot of what you say in so it’s good to have something written down.” (ID 22.2: SD, RA1, M)  “I think it is really important... one thing I’ve learnt actually over the years having been a patient a couple of times is that we bombard patients with information at point of discharge.. and they get home and they go “What did they say about the Panadol? Am I meant to have a headache?” My favourite one is where you discharge these poor people after they’ve had two Panadeine Forte and they don’t know what day it is. Like they’re a bit drowsy and you give them all this information and they don’t remember. So I’ve learned just through clinical experience as well as being a patient it is very important to write the stuff down.” (ID 22.5: SN, RA1, M)  “I think it is important because the problem is, you tell people things and they forget 90%, you know there’s studies that show people forget a lot of what they’re told anyway, so it would be good to do it as prompt, they can look at it afterwards.” (ID 19.3: SD, RA1, L)   “The most critical function of all of that is that people don’t worry about a symptom that they’ve got 2 or 3 days down the track and come back... certainly adequate information at the time of discharge prevents representation.” (ID 24.5: D, RA2, L)  “...really useful to give them a handout, it saves you talking for 10 minutes and rehashing the same thing over and over and it gives the patient a reference point as well. I think most people tend to give as much information as they can because they know it will prevent representation.” (ID 25.3: SN, RA2, M)   “Certainly it is [important]. I can say it is bad patient care, number one and number two it affects the outcome of the head injury, if I’m not giving enough information, how will the patient understand? That is the patient’s right to have all the information and that the second is it is your duty of care to give all the information.” (ID 6.2: SD, RA1, M). |
| **Key domain** | **Themes** | **Dr** | **Nurse** | **Representative quotations** |
| **Environment context and resources** | Increasing pressure on ED staff to discharge patients quickly to free up beds and cannot focus on follow-up care.  ED has a large flow of casual and pool staff who do not get staff orientation and don’t know where the patient information is.    Find it difficult to locate the patient information sheets on the Intranet.  Printed patient information sheets often run out and are not replaced.  Patient information sheets are unhelpful, ambiguous and vague.    Patient information sheets are out of date and not easy to read.  No barriers in ED to discharging a patient without information.  Patient information sheets are available in print and available on the internet. | ✓        ✓    ✓     ✓           ✓ | ✓      ✓                      ✓  ✓ | “We do have a lot of pressure on us and we are pushed for those beds and it’s just like “Get them out, get them out”. We get so focussed on getting them out of the department that we don’t focus on the follow up care and that’s probably the main factor.” (ID 25.2: SN, RA2, M)  “In most EDs there’s a large flow of casual and pool staff who don’t get staff orientation. So they don’t know that it’s [patient information] there. The ANUMs are under such pressure they don’t get time to remind every nurse.” (ID 25.2: SN, RA2, M)  “We have a pro-forma document on the computer system, printable from all our computers but it might depend on whether the doctor is aware that it’s there and also just time pressures.” (ID 19.5: SD, RA1, L)  “[Patient information] is on the Intranet, it’s sometimes not easy to navigate through for those who don’t know it.” (ID 19.3: SD, RA1, L)   “They should be outside on the board…but invariably they’re gone and somebody hasn’t replaced them so I have to go and print it out myself.” (ID 22.2: SD, RA1, M)    “Well, the [patient information sheets] that was sent around and told to replace all others, I found were pretty unhelpful. I thought it was a bit ambiguous. I don’t think we’re using it. They’re just a bit vague about what to do, when to come back. I just use it as a framework and just add to it.” (ID 25.5: SD, RA2, M)  “I think [it would be useful to have] some up to date information in a simple easy to read format ... it looks quite old the stuff we give out and we’re just going through a process of updating stuff and as soon as I see something that’s got 2002 written on it, I am not using it I like to see copyright 2011 on things...you feel much better giving them out when you know it’s up to date information.” (ID 25.3: SN, RA2, M)  “You can leave without having it. There’s nothing that you have to do, there’s no barrier for them going without it, so therefore it will get forgotten at times.” (ID 18.1: D, RA1, M)  “We’ve got printed copies...we’ve got the [organisation name] one, they are quite good and the ones from the [organisation name] are quite good. Whatever’s handy. If we run out of printed copies I just go to whatever website I can get up quickly and I will print. I think there was a hospital one still kicking around but on the whole now we’ve got so many electronic...that comes from a time when we didn’t have electronic references so I’d make sure that they get the information sheet.” (ID 22.5: SN, RA1, M) |
| **Key domain** | **Themes** | **Dr** | **Nurse** | **Representative quotations** |
| **Social /professional role and identity** | Providing patient information is within the doctor’s role. It should be a joint responsibility with nurses.  Would not delegate the role of providing patient information to the nurses however would occasionally delegate the role to residents or interns and show them how to do it.  In hospitals with a high number of rotating doctors, the role of provide the patient information is the nurses’ due to the doctors not knowing where the information sheets are.  Belief that nurses are better than doctors at providing patient information as they are more vigilant about printing out the written patient information and giving it to the patient.  Providing patient information is a central part of the ED discharge process. | ✓          ✓                   ✓         ✓ | ✓ | “[Providing the patient information] – it’s not necessarily the doctor’s role but I think it falls under the doctor’s role because a lot of our nurses won’t do it or don’t do it. Because they’re not experienced enough to be able to do it...it should be something that should be a joint responsibility...I think the problem is there is a lot of new junior nurses, they are very busy and pressed like everyone else.” (ID 19.3: SD, RA1, L)  “Absolutely it’s my role...if the nurse gets the chance she will explain all of these things. With me being the treating doctor it’s my responsibility. I make sure the nurse has told them or myself has told them. Sometimes it is doubling up.” (ID 6.2: D, RA1, M).  “I would definitely do that myself. I don’t delegate that to the nurses. I would occasionally delegate it to the primary treating doctors supposing one of my residents or interns had seen it and they may not even be aware of it. I would print it out for them, I’d show them and then I’d say go and discuss this with the patient.” (ID 4.2: SD, RA1, L)      “Because we have a high number of rotating doctors they wouldn’t know that the form was there. It would rely on the nursing staff saying “Oh, and I’ll just give them this information”... whose ultimate role would it be? It’s probably medical, they’re the ones that are ensuring that they’re safe to go home but then it sort of crosses a little bit over into patient advocacy on behalf of the nurse and ensuring the patient is fully informed.” (ID 37.3: SN, RA3, S)  “I think the nurses are probably better at it than the doctors. So they’d probably be more vigilant about printing out the information and making sure it’s given to the patients.” (ID 19.5: SD, RA1, L)       “I think written information is well supported as a central part of the Emergency Department discharge process because of the fact that people either are too stressed to remember what they’re told or like most of us can forget things they’re told. Or have further questions that they meant to ask but didn’t ask at the time which may be answered in the written documentation.” (ID 19.5: SD, RA1, L) |
| **Key domain** | **Themes** | **Dr** | **Nurse** | **Representative quotations** |
| **Memory, attention and decision processes** | Sometimes forget to provide patient information if the ED is busy and there are time and work pressures. | ✓ | ✓ | “Three or four out of ten patients would probably get the information sheet. Only because most of the time they’re either admitted or we just forget about it when they go home.” (ID 25.2: SN, RA2, M)  “Look definitely I forget about it, we’re all humans. Most likely if I am busy with another patient and the nurse comes and tell me “Look this patient is okay, can he go?” It happened the other day where I was busy with a trauma patient. And then actually after 1 hour I remember “Oh I didn’t give her the patient information.” (ID 24.2: D, RA2, L)  “I probably would not forget, I am a believer that there are other things that we often forget and whether I always remember to document that I gave them that then probably not. I would probably forget [to document] depending on how busy I am. I always make sure they at least go home with some plan. It’s one of those things that I think was drummed into us early that for something like [head injury] they need the discharge advice more than a lot of other conditions.” (ID 4.2: SD, RA1, L).  “It just gets missed. It wouldn’t be deliberately. If you are taken away to care for a very unwell person, they may have gone home without it and sometimes they leave it on the bedside.” (ID 39.2: N, RA2, S) |
| **Key domain** | **Themes** | **Dr** | **Nurse** | **Representative quotations** |
| **Knowledge** | Some clinicians are not aware patient information sheets are available.  Junior staff are not aware of the consequences of not providing the patient information. |  | ✓     ✓ | “I think a lot of the people don’t know the information sheets are there.” (ID 25.2: SN, RA2, M)    “Junior staff not so much...we try and encourage [the provision of written patient information] but I suppose they’re not aware of the consequences that can happen really...but I suppose until they’ve kind of experienced a patient quite unwell I don’t think that they’d probably get it.” (ID 37.2: N, RA3, S) |
| **Key domain** | **Themes** | **Dr** | **Nurse** | **Representative quotations** |
| **Beliefs about capabilities#** | Feel confident in accessing the patient information on the computer system and it takes little time to give it out. | ✓ |  | “...the information’s very easy to access. We have a proforma on the computer system, printable from all our computers but it might depend on whether the doctor is aware that it’s there and also time pressure. Not that it takes much time to print out and give it out.” (ID 19.5: SD, RA1, L) |

# Domain deemed as relevant but not a ‘key’ domain.

Table S4: Factors thought to influence the practice of providing brief, routine follow-up consisting of advice, education and reassurance

| **Key domain** | **Themes** | **Dr** | **Nurse** | **Representative quotations** |
| --- | --- | --- | --- | --- |
| **Environment context and resources** | ED sees too many patients to follow-up patients with mTBI.  With existing staffing levels and services, the ED cannot follow-up patients with mTBI.   ED not in a position to deal with sequelae or long-term problems.  Patient unlikely to see same doctor in ED for a scheduled follow-up, creating documentation and communication problems.  No referral mechanisms or pathways available in ED to refer a patient.    Some ABI services are available for ED to refer patients with mTBI for follow-up. | ✓      ✓    ✓       ✓      ✓ | ✓ | “We see way too many patients a day to even consider it. Once they leave here well that’s it, the next lot through. We have too many things to worry about.” (ID 25.3: SN, RA2, M)   “Formal cognitive assessment in the ED isn’t going to happen with the existing ED staff. Access to that sort of assessment next working day is probably the best you’re going to do and we don’t actually have that...not really suited to an acute hospital setting, particularly one that doesn’t have a neurosurgical neuropsych service...if we had people here we could refer to we could easily add that into routine follow-up.” (ID 25.5: SD, RA2, M)   “That’s the problem with ED, we’re not really in a position to deal with sequelae or potential long-term problems very well.” (ID 25.5: SD, RA2, M)   “Scheduled follow up, the main limitation is that the patient’s likely to see a different doctor from the one that they first saw. So that creates problems in terms of documentation, communication, comparison of critical findings.” (ID 19.5: SD, RA1, L)     “You can’t get follow-up in a place like this. It’s pretty hard. There are a couple of people that I really tried to [refer] but it was very difficult. There was no one who really was in that area of expertise. There’s no referral option at all. There’s probably someone in the organisation who might be interested in seeing them but certainly not at a systemic level. There’s no pathway to check though.” (ID 25.6: SD, RA2, M)  “You’d have to ask our brain injury nurse…she was begging for business. She found that most of the referrals that were coming from us [ED] they’d all been told everything they need to be told...and then she’d get these people who the damage had been done, who had gone back to work early, who were still getting headaches three to six months later and whose personality had changed a bit, who started as inpatients.” (ID 10.1: SD, RA1, M)  “As it currently stands, anyone who has a CT scan are referred to our acquired brain injury clinic.” (ID 24.5: D, RA2, L) |
| **Key domain** | **Themes** | **Dr** | **Nurse** | **Representative quotations** |
| **Social /professional role and identity** | ED clinicians do not have an active role in follow-up of these patients. Role of ED is to identify any life-threatening injuries and identify appropriate place for follow-up. | ✓ |  | “I suppose our role is to make sure that there isn’t a significant head injury and that their life’s not in threat and that would be, you know, you could end your responsibilities there but obviously I do worry about whether they would need the appropriate follow up and who should we be sending it to, you know, neuro rehab or neurologists and that sort of thing.” (ID 4.2: SD, RA1, L)  “I don’t think that we have an active role in follow-up. We deal with emergencies not with follow-up of things that haven’t happened. If there was a little bleed that became a big bleed and they were really sick that’s really not active follow-up its more they have deteriorated and they need emergency care now...I think our role is very much that for follow-up of patients to identify the most appropriate place to follow them up. At the moment we are sending all of these ones back to the GP, if we found that there was a better place [it might be different]...” (ID 4.4: SD, RA1, L).   “I don’t think myself or many people give much further thought to what happens afterwards.” (ID 19.4: SD, RA1, L) |
| **Key domain** | **Themes** | **Dr** | **Nurse** | **Representative quotations** |
| **Knowledge** | Do not know the number of patients with mTBI that reoccur or represent with ongoing problems.          Aware that some patients with mTBI have problems but do not know the link between the severity of the mild head injury and resulting problems.  Do not know how to organise a neuropsych review or if these services are available. | ✓              ✓        ✓ | ✓                      ✓ | “To be honest, I don’t know what the reoccurrence or representation with ongoing problems is. I don’t know if there’s a certain amount of small bleeds we’re missing.” (ID 22.5: SN, RA1, M)  “I wouldn’t know how many people really have mild injuries that might have some ongoing problems. I don’t know what the answer is...personally I don’t see it’s a huge burden on society, but the true ones are.” (ID 25.6: SD, RA2, M)  “I understand there can be issue in that context. I’ve never seen what happens to patients subsequently in terms of their effects. I wouldn’t be surprised if there are some effects. I’ve had one incident that comes to mind from years ago where a guy got a relatively mild concussion but was dramatically affected for quite awhile afterwards. I know they speak about concentration, moods all sorts of things. It can go on for weeks”. (ID 19.4: SD, RA1, L)  “We’re certainly aware of ABI problems, but making the connection, I suppose, between the two and maybe how minor the original injury appeared to be, no, and it is often minor. If they don’t have obvious personality change and cognitive defect but still have lost something, we’re not going to notice that...I don’t think there is that massive awareness.” (ID 25.5:SD, RA2, M)  “I don’t think I’ve ever specifically organised neuropsych review, I don’t know how to do it in this place. Or if it’s even available, or a psychologist or anything. I think because in this place it would be rarely required because the ones that are a big deal would be shipped off somewhere else. So the mild ones like I said go home so I am not aware specifically of that sort of head injury follow up.” (ID 19.4: SD, RA1, L)  “We don’t know how to do it...it just doesn’t get done. I haven’t seen an OT in the department for the whole time I’ve been here, so we don’t know they are around.” (ID 25.2: SN, RA2, M) |
| **Key domain** | **Themes** | **Dr** | **Nurse** | **Representative quotations** |
| **Beliefs about consequences** | Referring the patient to the GP reduces representations to the ED  Referring a patient with mTBI to the GP will result in a long waiting time and expense to the patient. Prefer that they represent to the ED.  Patients representing to the ED rather than the GP provide security to the treating ED clinician.  Referring patients to primary care and GPs is the fallback and safety net for the ED clinician.  Consequence of referring patients to a clinic is that they may flood the clinic with patients.  Believe it is important to follow up these patients to make sure they have no ongoing symptoms. | ✓       ✓  ✓     ✓ | ✓  ✓ | “It’s good enough from my perspective... I don’t want 30 footballers from the weekend coming back in on Tuesday to be reviewed in the emergency department. As far as being clinically appropriate I don’t know to be 100% sure.” (ID 25.3: SN, RA2, M)   “My preference is that if they’re following up head injuries I think that they come back here because all of the notes are here and we’re more used to dealing with it. There’s often a 2 to 3 week wait for patients to see their own GPs and a lot of these people don’t have private health insurance and don’t want to pay to see a doctor and basically there’s one place to get bulk-billed in town. So I mean people just come here...it’s quicker than going to see a GP and it’s cheaper.” (ID 24.4: D, RA1, L)  “If they’re still worried then I’ll probably just ask them to come back here instead of going to the GP...for my own security I would prefer to just go over the whole neurological examination at the same time as well.” (ID 39.3: D, RA2, S)  “For now we always rely on primary care, the GPs, at least, is a fallback and a safety and they won’t let someone they’re worried about just wander off.” (ID 4.2: SD, RA1, L)    “We certainly haven’t been sending everybody. I mean, we would be concerned about flooding a clinic if we send everyone who seemed to be okay just to be sure.” (ID 25.5: SD, RA2, M)    “I think it’s really important that they’re followed up... make sure they’ve no other ongoing symptoms and sometimes patients come in with one thing and realise there are other medical issues that have caused the head injury...medication related or something else.” (ID 4.3: N, RA1, L) |
| **Key domain** | **Themes** | **Dr** | **Nurse** | **Representative quotations** |
| **Motivation and goals** | Would be motivated to identify patients with potential long term problems if there was some way of following them up and helping them in a meaningful way. | ✓ |  | “If there were some way of following up or there was some way of actually helping that patient in a meaningful way other than the information that you can give them there at the time, then I think that would be quite motivating to find these people and look a bit harder for them...when there’s nothing to do for them, then there’s zero motivation.” (ID 25.6: SD, RA2, M) |

SD = senior doctor incl. consultant/ director, D = doctor incl. senior registrar, registrar, higher medical officer, SN = senior nurse incl. nurse practitioners, N = nurse
RA1 = Metro, RA2 = Inner Regional, RA3 = Outer Regional
S = small (<20000 presentations), M = medium (20,000 – 50,000), L = large (50,000+)
